# A gene expression signature of emphysema-related lung destruction and its reversal by the tripeptide GHK

**DOI:** 10.1186/gm367

**Published:** 2012-08-31

**Authors:** Joshua D Campbell, John E McDonough, Julie E Zeskind, Tillie L Hackett, Dmitri V Pechkovsky, Corry-Anke Brandsma, Masaru Suzuki, John V Gosselink, Gang Liu, Yuriy O Alekseyev, Ji Xiao, Xiaohui Zhang, Shizu Hayashi, Joel D Cooper, Wim Timens, Dirkje S Postma, Darryl A Knight, Marc E Lenburg, James C Hogg, Avrum Spira

**Affiliations:** 1 Division of Computational Biomedicine, Department of Medicine, Boston University School of Medicine, 72 East Concord Street, Boston, MA 02118, USA; 2 Bioinformatics Program, Boston University, 44 Cummington Street, Boston, MA 02215, USA; 3 UBC James Hogg Research Centre, Providence Heart + Lung Institute, St. Paul's Hospital and Department of Pathology and Laboratory Medicine, University of British Columbia, 1081 Burrard St, Vancouver, BC V6Z 1Y6, Canada; 4 Department of Pathology and Medical Biology, University Medical Center Groningen, University of Groningen, Hanzeplein 1, 9713 Groningen, Netherlands; 5 Department of Pathology and Laboratory Medicine, Boston University School of Medicine, 72 East Concord Street, Boston, MA 02118, USA; 6 Hospital of the University of Pennsylvania, Division of Thoracic Surgery, 3400 Spruce Street 6 White Building, Philadelphia, PA 19104, USA; 7 Department of Pulmonary Diseases, University Medical Center Groningen, University of Groningen, Hanzeplein 1, 9713 Groningen, Netherlands

## Abstract

**Background:**

Chronic obstructive pulmonary disease (COPD) is a heterogeneous disease consisting of emphysema, small airway obstruction, and/or chronic bronchitis that results in significant loss of lung function over time.

**Methods:**

In order to gain insights into the molecular pathways underlying progression of emphysema and explore computational strategies for identifying COPD therapeutics, we profiled gene expression in lung tissue samples obtained from regions within the same lung with varying amounts of emphysematous destruction from smokers with COPD (8 regions × 8 lungs = 64 samples). Regional emphysema severity was quantified in each tissue sample using the mean linear intercept (Lm) between alveolar walls from micro-CT scans.

**Results:**

We identified 127 genes whose expression levels were significantly associated with regional emphysema severity while controlling for gene expression differences between individuals. Genes increasing in expression with increasing emphysematous destruction included those involved in inflammation, such as the B-cell receptor signaling pathway, while genes decreasing in expression were enriched in tissue repair processes, including the transforming growth factor beta (TGFβ) pathway, actin organization, and integrin signaling. We found concordant differential expression of these emphysema severity-associated genes in four cross-sectional studies of COPD. Using the Connectivity Map, we identified GHK as a compound that can reverse the gene-expression signature associated with emphysematous destruction and induce expression patterns consistent with TGFβ pathway activation. Treatment of human fibroblasts with GHK recapitulated TGFβ-induced gene-expression patterns, led to the organization of the actin cytoskeleton, and elevated the expression of integrin β1. Furthermore, addition of GHK or TGFβ restored collagen I contraction and remodeling by fibroblasts derived from COPD lungs compared to fibroblasts from former smokers without COPD.

**Conclusions:**

These results demonstrate that gene-expression changes associated with regional emphysema severity within an individual's lung can provide insights into emphysema pathogenesis and identify novel therapeutic opportunities for this deadly disease. They also suggest the need for additional studies to examine the mechanisms by which TGFβ and GHK each reverse the gene-expression signature of emphysematous destruction and the effects of this reversal on disease progression.

## Background

Chronic obstructive pulmonary disease (COPD) is a significant public health problem worldwide and the third leading cause of death in the United States [1]. It is characterized by irreversible airflow limitation due to obstruction in the small conducting airways and emphysematous destruction of the gas exchanging tissue of the lung. Tobacco smoke is a significant risk factor for COPD and at least 25% of smokers develop this disease [2]. Current theories concerning disease pathogenesis include an imbalance between protease and anti-protease activity, induced apoptosis of alveolar cells through deregulation of pathways involved in oxidative stress, chronic inflammation, and aberrant tissue remodeling that lead to the destruction of the extracellular matrix (ECM) in the lung [3,4]. Lung repair and regeneration are potential processes to target with novel therapeutics in COPD as abnormal tissue repair by the epithelial-mesenchymal trophic unit can result in either fibrosis or destruction of the ECM [5]. However, the molecular mechanisms responsible for the pathogenesis of COPD remain poorly understood.

Several groups have profiled gene expression in patients with and without COPD or between patients with varying levels of airflow obstruction in order to understand differences in gene expression related to COPD [6-11]. While these studies have provided an initial look into the COPD transcriptome, their results primarily relied on the use of lung function tests to define the presence or degree of COPD. Lung function phenotypes can neither distinguish between obstruction in the small airways and emphysematous destruction of the lung parenchyma nor provide information about regional differences in disease severity. Recently, McDonough *et al. *used micro-CT scans to quantify the degree of emphysema in different regions of lungs from patients with severe COPD by measuring the mean linear intercept (Lm), a morphological measurement of alveolar destruction [12]. In order to gain insights into biological pathways associated with increasing emphysema severity within a patient and explore computational strategies for identifying COPD therapeutics, we obtained paired samples from eight regions at regular intervals between the apex and base of each explanted lung from six patients with severe COPD (Global Initiative for Chronic Obstructive Lung Disease (GOLD) stage IV) and two donor lungs. The degree of emphysematous destruction was quantified in one tissue sample from each region by Lm, while gene expression was profiled in the adjacent tissue sample from the same region.

We identified a number of genes whose expression is associated with increasing emphysematous destruction and found that pathways enriched among these genes were involved in the immune response and tissue remodeling. Using the Connectivity Map (CMap) [13], we found that the tripeptide Gly-His-Lys (GHK) was able to reverse the aberrant patterns of gene expression associated with increasing emphysema severity and induce patterns of gene expression consistent with transforming growth factor beta (TGFβ) pathway activation. Furthermore, we showed that by treating distal lung fibroblasts from COPD patients with GHK, we can restore normal contractile function through re-organization of the actin cytoskeleton and up-regulation of integrin-β1. These data further support the potential of GHK as a therapeutic in the treatment of emphysema.

## Materials and methods

### Sample acquisition and processing

Single lungs (*n *= 6) were removed from patients treated for severe COPD by double lung transplantation at the University of Pennsylvania. Donor lungs (*n *= 2) for which no suitable recipient was identified were released for research use from the Gift of Life Organ Procurement Organization in Philadelphia. This study was approved by the institutional review boards and conforms to the Helsinki Declaration. Written informed consent for use of these specimens and the relevant clinical and radiological data required for this research were obtained from each patient prior to surgery and from the next of kin of the persons whose donated lung was released for research. Each lung was removed from the thorax, cooled to 1.6°C, and transported to the laboratory where the bronchial stump was cannulated [14]. The lung was then inflated using a compressed air source attached to an underwater seal to slowly increase transpulmonary pressure (PL) from 0 to 30 cmH_2_O. The specimen was then held at a transpulmonary pressure of 10 cmH_2 _Owhile frozen by liquid nitrogen vapor (-130°C). The frozen specimen had a multidetector CT scan followed by being cut into 2-cm thick slices in the same plane as the CT scan. Tissue samples were collected using a sharpened steel cylinder (cork bore diameter of 14 mm). One sample from a cluster of four core samples of lung obtained from each site was processed for micro-CT [12]. A companion core from the same cluster was used for the gene profiling and validation studies reported here. The representative nature of these samples with respect to the entire lung was established by comparing the densities of the sampled sites with the frequency distribution of the densities in the entire lung on multidetector CT as reported in McDonough *et al*.[12].

### Measurement of mean linear intercept

The severity of emphysema within each core was estimated by measuring Lm. A micro-CT scan of each core provided approximately 1,000 contiguous 16-μm thick images. Lm was measured at 20 regularly spaced intervals of each of the micro-CT scans using a previously validated grid of test lines projected onto the image and a custom macro linked to specialized software (ImagePro Plus; MediaCybernetics (Rockville, MD, USA). The number of intercepts between these lines and tissue was counted. Lm was calculated as the total length of the test lines divided by the number of cross-overs with tissue (equal to the number of intercepts divided by 2).

### Microarray sample processing

High molecular weight (mRNA-containing fraction) RNA was isolated from tissue cores using the miRNeasy Mini Kit (Qiagen, Valencia, CA, USA). RNA integrity was assessed using an Agilent 2100 Bioanalyzer and RNA purity was assessed using a NanoDrop spectrophotometer. RNA (1 μg) was processed and hybridized onto the Human Exon 1.0 ST array (Affymetrix Inc., Santa Clara, CA, USA) according to the manufacturer's protocol as previously described [15]. Expression Console Version 1.1 (Affymetrix Inc.) was used to generate transcript-level gene expression estimates for the 'core' exon probesets via the robust multichip average (RMA) algorithm. Gene symbols of transcript IDs were retrieved using DAVID [16]. These gene expression data are available through the Gene Expression Omnibus (GEO) under the accession GSE27597.

### Microarray data analysis

Two linear mixed-effects models were used to identify gene expression profiles associated with the degree of regional emphysema severity as measured by Lm:

1. $\text{Gen}{\text{e}}_{ij}=\;\beta_{0}\;+\;\beta_{\text{Slice}}\;\times \;\text{Slic}{\text{e}}_{ij}\;+\;\alpha_{\text{j}}\;+\;\varepsilon_{ij}$

2. $\text{Gen}{\text{e}}_{ij}=\;\beta_{0}\;+\;\beta_{\text{Slice}}\;\times \;\text{Slic}{\text{e}}_{ij}\;+\beta_{\text{Lm}}\;\times \;\text{L}{\text{m}}_{ij}\;+\;\alpha_{j}\;+\;\varepsilon_{ij}$

i=1,2,...,8;j=1,2,...,8

$$\varepsilon_{ij}\;\sim \;N\left(0,\;\sigma^{2}\right)\;\alpha_{j}\;\sim \;N\left(0,\sigma_{a_{j}}^{2}\right)$$

Gene*_ij _*is the log_2 _expression value for sample *i *in patient *j *for a single gene. Slice is a fixed effect controlling for the position within the lung from which the sample core was obtained. The random term ε*_ij _*represents the random error, which was assumed to be normally distributed, α*_j _*represents the random effect for patient, and β_0 _represents the intercept. Model 2 contains an additional fixed effect term for emphysema severity measured by the natural log of Lm. A gene's expression profile was considered associated with Lm if model 2 fit better than model 1 as determined by a significant *P*-value from a likelihood ratio test between the two models after applying a false discovery rate (FDR) correction. In the immunohistochemistry experiments, these linear models were also used to examine the relationship between Lm and the volume fraction of tissue with positive staining by substituting volume fraction (Vv) for gene expression as a dependent variable. All statistical analyses were conducted using R statistical software v2.9.2 and the nlme package in Bioconductor v2.4 [17].

### Functional enrichment analysis

Functional enrichment analysis was performed using DAVID 2008 or Gene Set Enrichment Analysis (GSEA) v2.0.7 [16,18]. For DAVID, functional enrichment was examined among Gene Ontology categories, and KEGG and BIOCARTA pathways. All genes in the species *Homo sapiens *were used as a reference set. For GSEA, genes were ranked by the t-statistic of the β_Lm _coefficient in the linear mixed-effects model and then analyzed for the enrichment of canonical pathways and Gene Ontology term gene sets obtained from MSigDB v2.5.

### Connecting to other gene-expression datasets

Using GSEA, sets of genes reported to change with COPD-related phenotypes or with TGFβ treatment in other gene-expression studies were examined in a ranked list of genes ordered from most induced in severe emphysema to most repressed in severe emphysema by the t-statistic of the β_Lm _coefficient in the linear mixed-effects model. Conversely, sets of genes we identified as significantly positively or negatively associated with Lm were examined within gene lists ranked by the degree of differential expression as determined by re-analyzing previously published COPD-or TGFβ-related microarray studies. See Additional file 1 for a description of the data normalization procedures and statistical analyses used to generate gene sets and/or ranked gene lists for each of the previously published gene-expression datasets.

### Connectivity Map

In order to find compounds that reverse gene-expression patterns associated with emphysema severity, we generated separate signatures for each COPD or TGFβ gene-expression dataset examined in this study. Signatures were generated by identifying the 50 genes most up-regulated and the 50 genes most down-regulated with respect to a COPD or TGFβ-related phenotype. Each signature was queried against the CMap using the algorithm described by Lamb *et al*.[13]. See Additional file 1 for a description of the statistical analysis used to generate each query signature for each phenotype within each dataset. The list of all CMap query signatures used in this analysis include: 1) genes that change in expression as a function of regional emphysema severity in this study; genes that change in expression with 2) forced expiratory volume in 1 second (FEV_1_), 3) FEV_1_/forced vital capacity (FVC), or 4) between cases versus controls in Bhattacharya *et al*.[7]; genes that change in expression between 5) controls versus emphysema patients or between 6) controls versus α1-antitrypsin disease in Golpon *et al*.[6]; genes that change in expression with 7) FEV_1 _or 8) diffusing capacity of carbon monoxide (DLCO) in Spira *et al*.[9]; genes that change in expression with 9) FEV_1_, 10) FEV_1_/FVC, 11) DLCO, 12) non-smokers versus GOLD2, or 13) non-smokers versus GOLD3 in Wang *et al*.[10]; and genes that change in expression with TGFβ treatment from 14) Qin *et al*.[19], 15) Classen *et al*.[20], 16) Renzoni *et al*.[21], 17) Koinuma *et al*.[22], and 18) Malizia *et al*.[23]. For comparison of the CMap data to our *in vitro *studies of the effects of GHK in primary lung fibroblasts, raw data for GHK-treated and control samples were downloaded from the CMap website and normalized using MAS5.0 with the Affymetrix CDF. Genes were ranked by a paired *t*-test between treatment and controls of different batches and compared to gene sets of GHK and TGFβ treatment using GSEA.

### Isolation and culture of lung fibroblasts

Lung tissue of former smokers (defined as quitting smoking for at least one year before surgery) with normal lung function or GOLD stage IV COPD was obtained from patients undergoing surgery for resection for pulmonary carcinoma or lung transplantation. Fibroblast cultures were established from parenchymal lung tissue by an explant technique as previously described [24]. Isolated cells were characterized as fibroblasts by morphological appearance and expression pattern of specific proteins as described previously [24,25]. Fibroblast cultures were stored into liquid nitrogen until use.

### Immunofluorescence

Fibroblast cultures at passage 3 were cultured in eight-well chamber slides (Gibco, Burlington, ON, Canada) in growth medium (DMEM, 10% fetal bovine serum (FBS), penicillin, and streptavidin from Invitrogen, Burlington, ON, Canada). After reaching 70% confluence, fibroblasts were cultured for 24 h in 1% FBS DMEM and then incubated with either TGFβ1 10ng/ml (Peprotech, Dollard des Ormeaux, Quebec, Canada), GHK 10 nM (Sigma, Markham, Ontario, Canada) or control media (1% FBS DMEM, penicillin, streptavidin) for a further 48 h. After stimulations, chamber slides were fixed with 4% paraformaldehyde for 20 minutes, blocked in 10% goat serum in phosphate-buffered saline (PBS) with 0.1% saponin for 1 h and then stained with integrin-β1 antibody (M-106, Santa Cruz Biotechnology, Santa Cruz, CA, USA) in 0.1% saponin in PBS for 2 h at room temperature. Following washing in PBS with 0.1% saponin and 0.1% Tween 20, secondary antibody conjugated with goat anti-Mouse IgG Alexa Fluor 488 and Phalloidin conjugated with Alexa Fluor 594 were incubated for 2 h at room temperature. Following final washes, cultures were incubated with DAPI 1 ng/ml and then coverslipped with cytoseal. Confocal images were acquired with a Leica AOBS SP2 laser scanning confocal microscope (Leica, Heidelberg, Germany). The images were overlaid and the contrast enhancements were performed on the images using Volocity software™ (Improvisions Inc., Boston, MA, USA) as previously described [26].

### Collagen gel contraction assays

Fibroblast cultures at passage 3 were cultured in six-well tissue culture plates (Gibco, Canada) in growth medium (DMEM, 10% FBS, penicillin, and streptavidin from Invitrogen, Canada). After reaching 70% confluence, fibroblasts were cultured for 24 h in 1% FBS DMEM and then incubated with TGFβ1 10 ng/ml (Peprotec, Canada), GHK 10 nM (Sigma, Canada) or growth media control for a further 48 h. Prior to the end of the treatment time point, a 12-well tissue culture plate was incubated with 1% bovine serum albumin in DMEM for 2 h. The medium was removed and then 500 µl of 0.4 mg/ml type I collagen (BD Biosciences, Mississauga, ON, Canada) was added and allowed to polymerize for 8 h at 37°C. The treated fibroblasts were then trypsinized and seeded at 2 × 10^5 ^cells/500 µl of 1% FBS DMEM, penicillin, streptavidin in duplicate on the collagen gels and cultured for an additional 24 h at 37°C in 5% CO_2_. The gels were imaged before and after and the extent of gel contraction measured using Image Pro Software.

### Multi-photon and second harmonic generation microscopy

The collagen gels were fixed with 4% paraformaldehyde for 20 minutes and washed in PBS with 0.1% saponin and 0.1% Tween 20 before being incubated with phalloidin conjugated with Alexa Fluor 594 for 1 h at room temp. Gels were then mounted on to a glass slide using Secure-seal™ imaging spacers (size 20 mm; Sigma) and aqueous mounting media. The gels were then imaged using second harmonic generation microscopy to determine fibrilar collagen as previously described [27]. For each cell volume, Z-section images were compiled and the three-dimensional image restoration was performed using Volocity software (Improvisions, Inc.). A noise-removal filter with a kernel size of 3 × 3 was applied to these three-dimensional images.

## Results

### Study population

**Figure 1:**
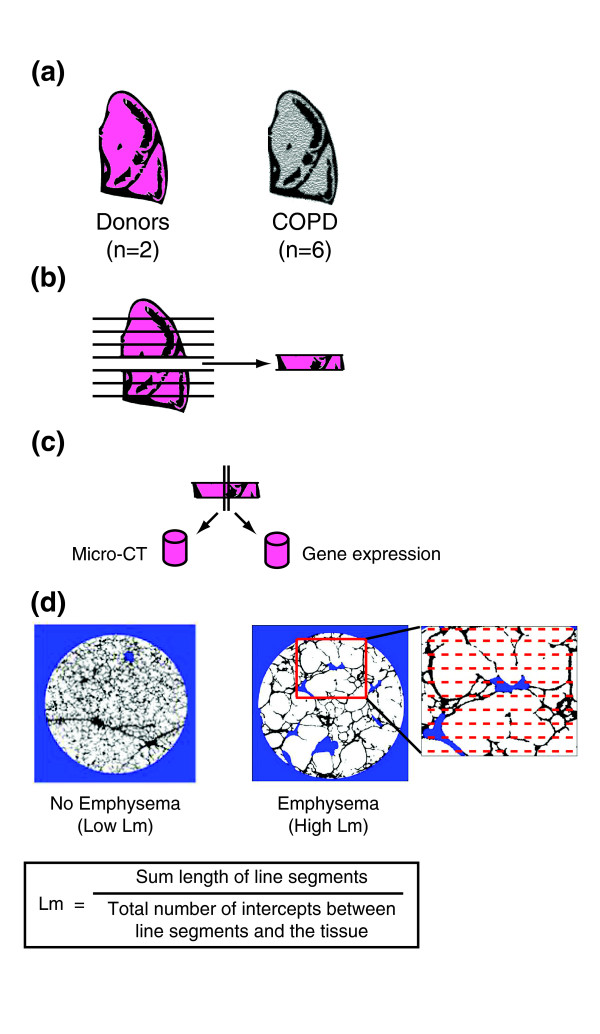
**Outline of study design**. **(a) **Whole lungs were removed from patients with severe COPD and from donors, inflated with air, and rapidly frozen in liquid nitrogen vapor. **(b) **The frozen specimens were cut into 2-cm slices from apex to base of the lung. **(c) **Adjacent tissue cores were removed from 8 different slices of each lung (8 patients with 8 slices = 64 total regions). **(d) **Micro-CT was used to measure Lm at 20 evenly spaced intervals throughout one core from each region.

Lm was quantified using micro-CT scans in eight samples taken at regular intervals from apex to base of lungs from six subjects that required transplantation for COPD and two organ donors (Figure 1). Table 1 shows demographic information and clinical characteristics of the eight subjects used in this study. As expected, samples from subjects with COPD had a higher mean and a greater range of Lm values between samples compared to those from donor lungs, indicating that there are regions of severe emphysema in COPD subjects (Table 1). Subject 6967 was diagnosed with a pure airway obstruction COPD phenotype without emphysema [28]. Consistent with this diagnosis, the distribution of Lm measurements for this patient closely resembles the distribution of Lm measurements from the donor lungs. Subject 6970 was diagnosed with α1-antitrypsin deficiency. The remaining four subjects with COPD had the centrilobular emphysematous phenotype commonly observed in smokers. The distribution of emphysematous destruction in tissue cores from these COPD patients range from little to no emphysema (Lm < 600) to very severe emphysema (Lm > 1,000) [12]. Subject 6969 had one sample excluded from subsequent analysis because its Lm measurement was an outlier (more than three times the interquartile range of the distribution of Lm measurements in all cores from all lungs examined).

**Table 1:** Subject demographics for lung tissue samples

Patient ID	Description	Sex	Age	Pack years	Smoking status	Lm mean ± SD (μm)	Lm range (μm)
6965	COPD	M	62	50	Former	716 ± 164	494-982
6967	COPD	F	61	25	Former	414 ± 82	334-585
6968	COPD	F	63	38	Former	724 ± 252	357-1,013
6969	COPD^a,b^	F	56	54	Former	1,822 ± 1270	521-4,620
6970	COPD^c^	M	55	15	Former	1,352 ± 599	647-2,551
6971	COPD	M	59	30	Former	1,097 ± 441	720-2,101
6982	Donor	M	59	-	Never	384 ± 47	344-473
6983	Donor	M	62	24	Former	289 ± 41	231-352

Subjects with COPD had FEV_1_/FVC <70% and FEV_1 _<25% predicted. ^a-c^Some patients had other diseases: ^a^von Willebrand disease; ^b^hypertension; ^c^α1-antitrypsin deficiency disease.

### Pathways associated with regional emphysema severity

**Figure 2:**
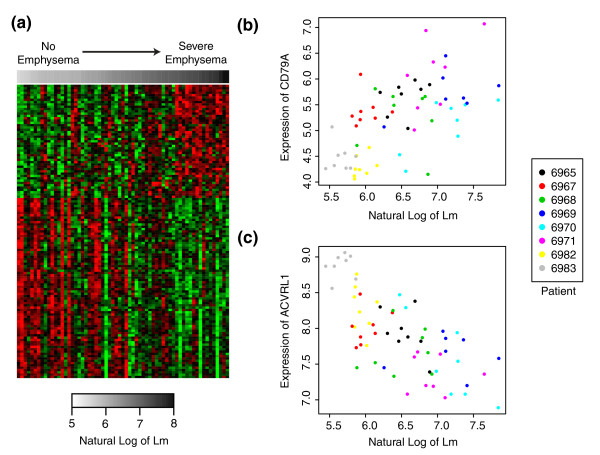
**Gene expression signature of regional emphysema severity**. **(a) **Supervised heatmap of genes whose expression is associated with Lm (FDR <0.10). Samples are organized from low to high Lm. Each row corresponds to a gene and each column corresponds to a sample. Green represents lower relative expression and red represents higher relative expression. **(b,c) **Expression of CD79A (b) and ACVRL1 (c) are plotted against the natural log of Lm with the color of each point indicating the subject from which the sample was derived.

Using linear mixed-effect models, the expression levels of 127 genes were significantly associated with Lm and thus associated with regional emphysema severity (Figure 2a; FDR <0.10; see Additional file 2 for the analytic results for all genes). Using DAVID [16] or GSEA [18], we found that genes with functions in the B-cell receptor signaling pathway were over-represented among the up-regulated genes, while genes involved in cellular structure, integrin signaling, extracellular matrix production, focal adhesion, blood vessel morphogenesis, and the vascular endothelial growth factor and TGFβ pathways were enriched among the down-regulated genes (FDR <0.05; see Additional file 3 for a list of all significantly enriched pathways). The expression of CD79A, a component of the B-cell receptor, increased in expression with increasing emphysema severity (Figure 2b), and the expression of ACVRL1 (also known as activin-like kinase I), a receptor in the TGFβ pathway, decreased in expression with increasing emphysema severity (Figure 2c). These two genes are shown as examples of the characteristic relationship between Lm and gene expression as observed in Figure 2a. To predict transcription factors that might be responsible for the observed patterns of differential expression, we inferred a gene expression relevance network using the Context Likelihood of Relatedness (CLR) algorithm [29]. Transcription factors with the most connections to other genes included EPAS1 (also known as HIF-2α), KLF13, TAL1, TBX3, GATA2, and BCL11A (Additional file 4). Fourteen genes whose expression is significantly correlated with regional emphysema severity or transcription factors that are highly connected to these genes in the relevance network were selected for quantitative RT-PCR validation in a subset of tissue cores from subjects with severe emphysema (see Additional file 1 for methods). Twelve out of the fourteen genes had a significant correlation between the expression values derived from the microarray and quantitative RT-PCR, showing that the association of gene expression with regional emphysema severity is reproducible across assays (Pearson correlation, *P *< 0.05; Additional file 5).

In order to demonstrate that the 127 gene signature is related to regional emphysema severity within individuals and not to differences between donors and COPD patients or to differences in levels of emphysema between COPD patients, we repeated the same statistical analysis while only including the five COPD patients with emphysema and standardizing the Lm measurements within each patient core to a mean of zero and a standard deviation of one (Z-score). Using GSEA, the sets of up-and down-regulated genes in the 127-gene signature identified in the previous analysis with all eight patients and unscaled Lm measurements were concordantly enriched among genes differentially expressed when only the five emphysema patients were analyzed with Z-scored Lm measurements, indicating that this gene signature is associated with regional emphysema severity (FDR <0.001, GSEA; see Additional file 6 for the enrichment plot).

### Validation of pathways up-regulated in regions of severe emphysema

**Figure 3:**
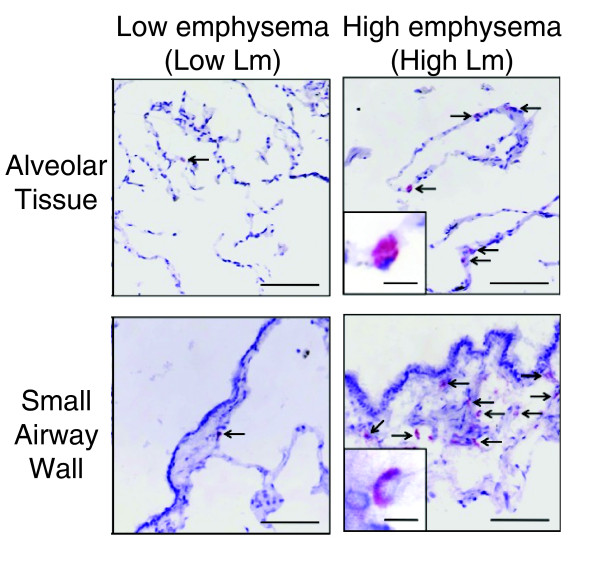
**Validation of differential expression for CD79A by immunohistochemistry**. Representative images of CD79A-positive cells (arrows) in the alveolar tissue and the small airway walls. Positive staining appears red. Scale = 200 µm; inset = 10 µm.

In order to investigate whether the up-regulation of components of the B cell receptor signaling pathway is associated with a change in the quantity of B cells in lung tissue, we quantified the Vv of CD79A protein, a marker for B cells, in relation to Lm by immunohistochemistry (see Additional file 1 for methods). CD79A-positive B cells were observed in the alveolar and small airway wall tissue (Figure 3). Vv was quantified in alveolar tissue for all 64 samples and in small airway tissue for 43 samples that contained small airways and was found to be positively correlated to Lm in both the alveolar and small airway wall tissue (*P *< 0.001), indicating that B cell abundance increases as emphysema severity increases.

### Validation of pathways down-regulated in regions of severe emphysema

**Figure 4:**
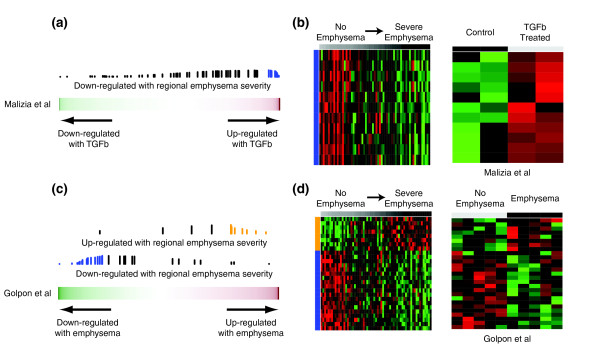
**Relation between gene expression changes associated with regional emphysema severity (Lm) and other gene-expression studies by GSEA**. **(a) **Relation between gene expression changes associated with regional emphysema severity and those induced by TGFβ treatment of A549 cells from Malizia *et al*.[23]. The color bar represents the fold change between the cell lines treated with and without TGFβ1for 11,910 genes in Malizia *et al*.[23]. Red indicates a more positive fold change and green indicates a more negative fold change (induced or repressed with TGFβ, respectively). The vertical lines represent the position of genes associated with regional emphysema severity in the ranked gene list. The height of the vertical lines corresponds to the magnitude of the running enrichment score from GSEA. Blue vertical lines indicate that the gene is part of the 'core' enrichment (that is, all the genes from the absolute maximum enrichment score to the end of the ranking). **(b) **Supervised heatmaps of relative gene expression levels for the core enrichment genes in both the regional emphysema and Malizia *et al*. datasets (11 genes down-regulated with emphysema severity but up-regulated with TGFβ). Each gene is represented in the same row across heatmaps. **(c) **Genes changing in expression with increasing regional emphysema severity were enriched in the cross-sectional study of COPD-related gene expression from Golpon *et al*.[6]. The color bar represents the t-statistic from a *t*-test between five emphysema patients and five non-smokers for 5,209 genes in Golpon *et al*.[6]. Blue and orange vertical lines indicate that the gene is part of the core enrichment. **(d) **Supervised heatmaps of relative gene expression levels for the core enrichment genes in both the regional emphysema and Golpon datasets (8 genes concordantly up-regulated; 19 genes concordantly down-regulated).

Several members of the TGFβ pathway were among the genes that had decreased expression as a function of regional emphysema severity. These genes included *ACVRL1, ENG, TGFBR2, *and *SMAD6*. Other components in this family, including *BMPR2 *(FDR q-value = 0.125) and *SMAD7 *(FDR q-value = 0.223), also showed evidence of modest down-regulation while *SMAD1 *showed evidence of modest up-regulation (FDR q-value = 0.101). To determine whether the TGFβ pathway might be affected by emphysema pathogenesis, we used seven previously published studies that had examined the effect of TGFβ ligands on gene expression to develop a collection of signatures of TGFβ pathway activation [19-23,30,31]. Genes that exhibited significantly decreased expression with increasing emphysema severity were enriched among genes induced in response to TGFβ treatment in a total of three datasets (FDR <0.05, GSEA). Similarly, the sets of genes most induced by TGFβ from each of the seven datasets examined were enriched among genes whose expression decreased as a function of emphysema severity (FDR <0.05, GSEA). As an example, the enrichment of genes associated with emphysema severity among genes changing with TGFβ treatment in the dataset from Malizia *et al*.[23] is shown in Figure 4a,b. See Additional file 7 for the GSEA enrichment plots for all seven datasets.

To further validate these findings, we cultured human lung fibroblasts with and without TGFβ1 and found that the set of genes most induced by TGFβ1 were enriched among genes that decrease in expression with increasing regional emphysema severity (FDR <0.05, GSEA; see Additional file 8 for the GSEA enrichment plot and Additional file 1 for the fibroblast culture methods). Immunostaining of lung tissue from the same regions on which we performed gene expression analysis localized SMAD2, a down-stream signal transducer of TGFβ, to the alveolar and airway walls while members of the bone morphogenetic protein (BMP) pathway, including SMAD6 and SMAD1, were primarily seen in vascular endothelial cells (Additional file 9).

### Relationship to expression profiles in other COPD studies

In order to show that the gene expression signature of regional emphysema severity is present in larger cohorts of patients with earlier stages of disease, we used GSEA to examine the relationship between genes associated with regional emphysema severity in this dataset and genes associated with COPD phenotypes in other cross-sectional studies [6-11]. The genes that decreased in expression with increasing emphysema severity were significantly enriched among genes down-regulated as a function of COPD-related phenotypes in four of the five previously published datasets that we examined (FDR <0.05, GSEA; Additional file 10). In addition, genes that increased in expression with increasing emphysema severity were enriched amongst genes up-regulated as a function of COPD-related phenotypes in three of the five datasets (FDR <0.05, GSEA). As an example, the enrichment of genes associated with regional emphysema severity among genes differentially expressed with the presence of emphysema in the dataset from Golpon *et al*.[6] is shown in Figure 4c,d. Conversely, sets of genes reported to be differentially expressed with COPD in four of the six other cross-sectional studies were enriched among the genes changing in expression with increasing regional emphysema severity (FDR <0.05, GSEA; Additional file 10). Examples of genes validated by quantitative RT-PCR in this study and concordantly differentially expressed in other studies are shown in Additional file 11. Many of these datasets, such as Bhattacharya *et al*.[7] and Wang *et al*.[10], contained larger numbers of patients with a variety of stages of disease (for example, GOLD stage 0 through GOLD stage IV). The enrichment of genes associated with regional emphysema severity with COPD-related phenotypes in these other datasets suggests that the biological processes associated with increasing emphysema severity within a patient with severe COPD also vary in individuals with earlier stages of disease.

### Prediction of novel therapeutics for emphysema

In order to identify compounds that might reverse the gene-expression pattern associated with progression of emphysema, we utilized the CMap [13], a compendium of microarray experiments that measure the effect of therapeutic compounds on gene expression in cancer cell lines. Signatures of genes that 1) change in expression with regional emphysema severity in this dataset, 2) change in expression with lung function measures in other datasets [6,7,9,10], or 3) change in expression with TGFβ treatment in other datasets [19-23] were each used as separate queries into the CMap data. We found that gene expression changes resulting from treatment with the tripeptide GHK, a compound thought to accelerate wound healing [32,33], were negatively correlated with expression patterns associated with increasing regional emphysema severity (*P *= 0.006) and the COPD-related expression patterns observed in Bhattacharya *et al*.[7] and Golpon *et al*.[6] (*P *< 0.05). In addition, the gene expression effects of GHK are similar to the effects of TGFβ treatment observed by Malizia *et al*.[23] (*P *= 0.004).

**Figure 5:**
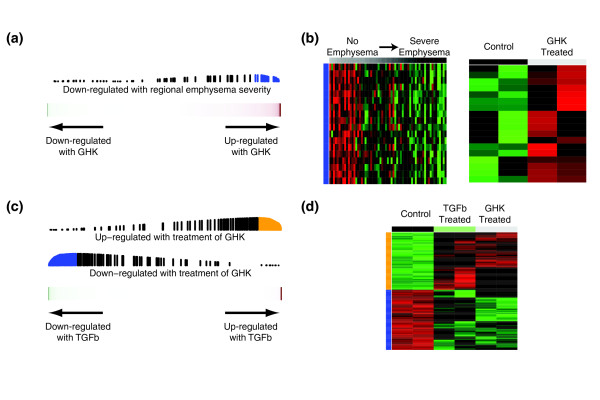
**Effect of GHK treatment on expression in human lung fibroblasts**. **(a) **Genes decreasing in expression with increasing regional emphysema severity were enriched among genes that are induced by GHK at 10 nM. **(b) **Supervised heatmaps of relative gene expression levels for the core enrichment genes in both datasets (18 genes down-regulated with Lm but up-regulated with GHK). Each gene is represented in the same row across heatmaps. **(c) **Genes differentially expressed with treatment of GHK at 0.1 nM were concordantly enriched among genes that change with treatment of TGFβ1. **(d) **Heatmap of relative gene expression levels for the core enrichment genes (118 genes up-regulated and 124 down-regulated with both GHK and TGFβ1).

As the CMap examined the effect of GHK in cancer cell lines, we next sought to verify the effect of GHK treatment in a cell type more relevant to emphysema pathogenesis. We utilized human lung fibroblasts because fibroblasts are the major interstitial cell within the alveolar unit that can synthesize and remodel the ECM and previous studies have demonstrated that GHK can induce ECM production in dermal fibroblasts [32-34]. Human lung fibroblast cultures were treated with two concentrations of GHK or with TGFβ1 (see Additional file 1 for methods). Gene expression profiling of these cells demonstrated that the 200 genes most induced by GHK at 1 μM in cancer cell lines in the CMap dataset were enriched among genes that increased after treatment with GHK at 0.1 nM in fibroblast cultures (FDR <0.05, GSEA). Furthermore, genes whose expression is decreased with increasing emphysema severity are enriched among genes induced by GHK at 10 nM (FDR < 0.05, GSEA; Figure 5a,b). Genes whose expression is altered by GHK treatment at either concentration are also enriched among genes that change with TGFβ1 treatment (FDR <0.05, GSEA; Figure 5c,d). See Additional file 8 for the GSEA enrichment plots showing the relationship between GHK, TGFβ, and emphysema severity signatures.

### Reversal of COPD-related phenotypes in fibroblasts by GHK

**Figure 6:**
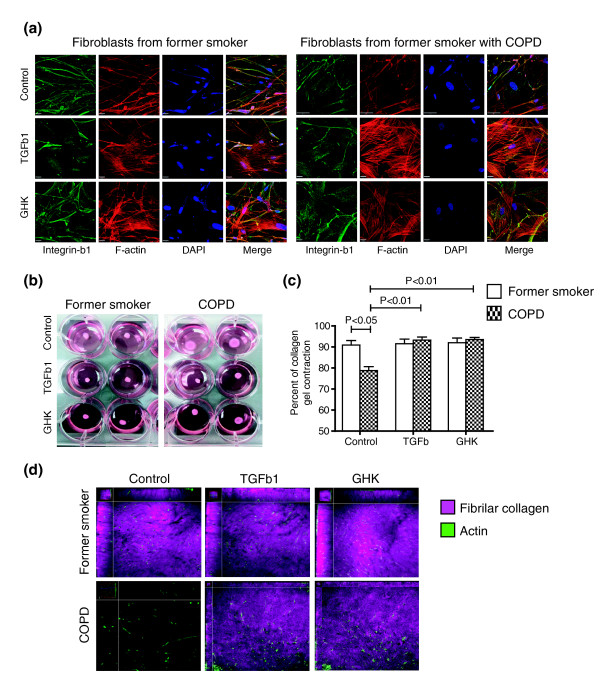
**Effect of GHK treatment on collagen contraction by fibroblasts from former smokers with COPD**. **(a) **Representative immunofluorescent images of distal lung fibroblasts from former smokers with and without COPD treated with GHK (10 nM), TGFβ1 (10 ng/ml), or media control for 48 h and stained with phalloidin to localize the actin cytoskeleton (red), integrin-β1antibody (green) and DAPI to localize nuclei (blue). **(b) **Representative images of collagen I gel bioassays at 24 h after being seeded with distal lung fibroblasts from former smokers with and without COPD previously treated with GHK, TGFβ1, or media control for 48 h. **(c) **The percentage of collagen I contraction was significantly decreased in fibroblasts derived from former smokers with COPD compared to former smokers without COPD (*P *< 0.05) but was significantly increased with addition of TGFβ1 or GHK (*P *< 0.01). **(d) **Representative enface Z-stack slices of three-dimensional reconstructed collagen I gel bioassays demonstrating actin in fibroblasts (green, phalloidin) and second harmonic signal originating from collagen fibrils (purple, 414 nM). Fibroblasts from former smokers with COPD were unable to efficiently remodel collagen into fibrils. However, this intrinsic defect was restored with treatment of TGFβ1 or GHK.

Genes induced in human lung fibroblasts after treatment with GHK were enriched in actin cytoskeleton organization and focal adhesion pathways (FDR <0.05, DAVID). These included integrins involved in collagen attachment, such as ITGB1. *ITGB1 *gene expression was also down-regulated with increasing emphysema severity in lung tissue (*P *= 0.008). Resolution of damaged tissue requires mesenchymal cells to attach to collagen fibers through integrin-dependent mechanisms and generate mechanical tension via the actin cytoskeleton to promote tissue contraction and wound size reduction. Using distal lung fibroblasts isolated from former smokers with and without COPD, we found that GHK (10 nM), like TGFβ1 (10 ng/ml), induced alterations in integin-β1 localization (green staining in Figure 6a) and reorganized actin to form contractile filaments (red staining in Figure 6a). We further demonstrated using a three-dimensional collagen gel contraction bioassay that distal lung fibroblasts derived from former smokers with COPD (*n *= 5) were unable to fully contract collagen I gels compared to fibroblasts obtained from former smokers without COPD (*n *= 5, *P *< 0.05; Figure 6b,c; see Additional file 12 for subject demographics), similar to what has been previously described [35]. However, fibroblasts derived from COPD patients first treated for 48 h with either TGFβ1 or GHK were able to induce full collagen I gel contraction comparable to that observed in fibroblasts from former smokers without COPD (*P *< 0.01, Figure 6b,c). Using the second harmonic generation properties of fibrilar collagen and multi-photon microscopy, we confirmed that fibroblasts from former smokers with COPD were unable to efficiently remodel collagen into fibrils (Figure 6d). Importantly, following 48 h of treatment with TGFβ1 or GHK on lung fibroblasts from former smokers with COPD, we were able to restore this intrinsic defect, which we propose is through organization of the actin cytoskeleton to a contractile phenotype as demonstrated by the confocal images displayed in Figure 6a.

## Discussion

The goal of this study was to identify gene expression changes associated with regional emphysema severity in order to elucidate biological processes underlying the progression of emphysema and to identify potential COPD therapeutics. By measuring gene expression from regions of varying emphysema severity within the same lung and by using a morphologic measurement of airspace size (Lm), which reflects the degree of alveolar destruction, we were able to identify gene expression changes associated specifically with the emphysematous component of COPD.

Interestingly, there was significant enrichment between genes differentially expressed in COPD or associated with worsening lung function in other datasets and those we found to be associated with regional emphysema severity. Importantly, this similarity supports the notion that regional differences in emphysema severity reflect the processes that occur with general COPD pathogenesis and progression and are not only present in patients with end-stage disease. Overall, these observations suggest a similarity in the gene expression alterations that accompany airflow obstruction, gas exchange abnormalities, and alveolar destruction measured by Lm.

A common characteristic in the pathology of COPD is progressive lymphocyte infiltration of the small airways and alveolar walls [36]. In addition, the formation of tertiary lymphoid organs within this infiltration suggests the presence of an adaptive immune response to persistent foreign or autoimmune antigens [37,38]. The present study extends these observations by showing that the expression patterns of several components of the B-cell receptor signaling pathway have increased expression in regions of severe emphysema. Igα (CD79A) and Igβ(CD79B) are proteins that associate with the B-cell receptor and transmit its signal upon stimulation. Immunohistochemistry showed a significant relationship between the volume fraction of the airway wall and alveolar tissue positively stained for CD79A and an increase in Lm. This relationship supports an increased number of B cells in both airway wall and alveolar tissues and is consistent with the induction of CD79A during tissue destruction associated with the increase in Lm.

The TGFβ signaling pathway is involved in a variety of cellular processes, including immune response, extracellular matrix remodeling, angiogenesis, and cell differentiation. This pathway has also been implicated in a variety of diseases such as cancer and fibrosis [39]. It has been hypothesized that the TGFβ pathway could play a role in COPD pathogenesis, but its role is not completely understood [40]. Togo *et al*.[35] found that fibroblasts isolated from COPD patients exhibited reduced chemotaxis, reduced nuclear to cytoplasmic ratios of phosphorylated SMAD3, and decreased α-smooth muscle actin production compared to controls when treated with TGFβ. Decreased mRNA expression or protein levels for TGFβ1, TGFBR1 [41], SMAD3 [42], SMAD6 [43], and SMAD7 [41,43] have been reported in more advanced stages of COPD or fibroblasts from COPD patients. In both alveolar and bronchiolar epithelium of emphysematous lungs, a decrease in phosphorylated SMAD2 has been shown by immunohistochemistry [44]. In normal human lung parenchyma, repair processes in response to mechanical injury are associated with increased TGFβ signaling, while a decrease in expression has been observed for TGFβ-related genes with worsening lung function in patients with COPD [25,45]. Furthermore, association studies have identified both promoter and coding region polymorphisms in the *TGFβ1 *gene that associate with increased risk for COPD [46-48]. In the present study, we identified several components of the TGFβ and BMP pathways that have decreasing expression with increasing emphysema severity. In the BMP pathway, ACVRL1 and ENG are receptors involved in the phosphorylation of SMAD1 and are expressed in the mature lung vasculature. The changing expression of SMAD6 and SMAD1, their localization predominantly to vascular endothelial cells, and the roles of ACVRL1 and ENG in angiogenesis support the hypothesis of aberrant tissue remodeling in the lung vasculature during emphysema pathogenesis. In the TGFβ pathway, TGFBR2 is a receptor involved in the phosphorylation of SMAD2/3 and is important for many tissue remodeling processes, including wound repair. Moreover, genes found to be induced by TGFβ in diverse studies were down-regulated in regions of severe emphysema. The localization of SMAD2 to alveolar and airway tissue and the decreased TGFβ pathway activity seen with increasing emphysema severity support the hypothesis that a decrease in TGFβ pathway activity also contributes to emphysema pathogenesis.

As COPD remains a major public health concern due to lack of effective therapeutic strategies, we sought to use computational methods to identify compounds that might modulate molecular processes associated with emphysema pathogenesis. The CMap is a large compendium of microarray experiments that measures the effect of over 1,000 compounds on gene expression in several cell lines [13]. By querying a gene expression signature of disease pathogenesis against the CMap dataset, one can find compounds that elicit a pattern of gene expression that is the opposite to the disease-related gene expression profile. This can lead to the hypothesis that such compounds, since they reverse the disease-related gene expression pattern, are potential therapeutics for that disease. This approach has been recently successful in the therapeutic repositioning of the antiulcer drug cimetidine to lung adenocarcinoma and the anticonvulsant drug topiramate to inflammatory bowel disease [49,50]. In these studies, signatures for each disease were derived using several publicly available gene-expression datasets and queried in the CMap. Candidate compounds or drugs that could significantly reverse the disease-related signatures of gene expression were further validated *in vitro*, showing that this computational method is a viable approach for identifying novel therapeutics.

Using the CMap dataset, we identified a relationship between the gene expression changes induced by the tripeptide GHK and those that are repressed with increasing emphysema severity. Intriguingly, we further found that GHK-treatment induced a pattern of gene expression similar to that resulting from TGFβ pathway activation. We replicated both of these findings in human lung fibroblasts, which are the major interstitial cells that maintain tissue structural integrity by sculpting the connective tissue. GHK-Cu is a natural tripeptide that, in human plasma, can be found at a concentration of 200 ng/ml at the age of 20 years but drops to around 80 ng/ml by the age of 60 years [34]. Characterization GHK-Cu in skin wound repair models suggests that it induces wound contraction, cell proliferation, angiogenesis, and increased expression of antioxidant enzymes and integrins [34,51]. Direct evidence for the ability of GHK-Cu to promote wound healing comes from experimental rat models where GHK treatment causes an acceleration of healing and a concentration-dependent increase of connective tissue and other ECM components [32,33]. These effects are consistent with the gene expression alterations induced by GHK and TGFβ treatment. Moreover, we confirmed these similarities by demonstrating that GHK and TGFβ induced significantly higher expression and re-organization of actin and integrin-β1 in distal lung fibroblasts.

We further assessed the ability of GHK and TGFβ to induce tissue contraction. As in previous studies [35], we demonstrated that distal lung fibroblasts derived from COPD patients have intrinsic defects in collagen I contraction compared to fibroblasts derived from former smokers without COPD. When fibroblasts from COPD lungs were treated with GHK or TGFβcontraction and remodeling of collagen gels was induced to levels comparable to fibroblasts from former smokers without COPD. We further demonstrated that the collagen contraction induced in COPD fibroblasts by GHK involves the organization of collagen I gels into collagen fibrils using multi-photon microscopy. Taken together, these data further support the hypothesis in which a wound-healing-like process is diminished as a function of emphysema progression and further suggest that this process is related to the TGFβ pathway.

While the number of subjects in this study for genomic analysis was small, the analysis of eight specimens per lung representing different degrees of emphysema from each individual allowed us to detect gene expression changes specifically associated with regional emphysema severity. We further demonstrated that these genes are concordantly differentially expressed in previous cross-sectional studies involving larger numbers of individuals with varying degrees of airflow limitation. These results validate the gene expression differences associated with regional emphysema severity in independent cohorts from different clinical settings and support the hypothesis that the genes whose expression is associated with regional emphysema severity reflect the activity of true disease-associated processes. As demonstrated by our micro-CT data, COPD is a heterogeneous disease within the lung [12]. Further studies will be required to assess whether COPD-associated differences in ECM remodeling by distal fibroblasts *in vitro *is associated with the regional disease severity in the tissue from which the fibroblasts are derived.

## Conclusions

This study has provided insights into molecular processes associated with emphysematous destruction of the lung and revealed mechanisms that contribute to the pathogenesis of COPD. Whole genome gene-expression analysis supports the role of the immune response in regional emphysema and elucidates additional pathways involved in the process of emphysematous destruction. The suggestion that progressive emphysematous destruction is associated with down-regulation of genes involved in or downstream of tissue remodeling and wound repair pathways supports a role for defects in ECM homeostasis and angiogenesis in the emphysematous destruction that occurs with chronic inflammation in COPD. We propose that these processes could be linked through decreased TGFβ pathway activation. These data are supported by our identification of GHK as a compound with the potential to mimic TGFβ pathway activity and induce collagen contraction, an important functional component of wound repair.

## Abbreviations

BMP: bone morphogenetic protein; CMap: Connectivity Map; COPD: chronic obstructive pulmonary disease; CT: computed tomography; DLCO: diffusing capacity of carbon monoxide; DMEM: Dulbecco's modified Eagle's medium; ECM: extracellular matrix; FBS: fetal bovine serum; FDR: false discovery rate; FEV_1_: forced expiratory volume in 1 second; FVC: forced vital capacity; GOLD: Global Initiative for Chronic Obstructive Lung Disease; GSEA: Gene Set Enrichment Analysis; Lm: mean linear intercept; PBS: phosphate-buffered saline; TGF: transforming growth factor; Vv: volume fraction.

## Competing interests

Boston University has intellectual property related to the work described in this manuscript.

## Authors' contributions

AS, JCH, MEL, DAK, DSP, and WT conceived and designed the experiments. JEM, TLH, DVP, CAB, MS, JVG, GL, YOA, JX, XZ, and SH performed the experiments. JDC, JEZ, TLH, DVP, and CAB analyzed the data. JDC and JCH contributed materials. JDC, MEL, JCH, and AS wrote the manuscript. All authors read and approved the final version for publication.

## Supplementary Material

Additional file 1 **Supplementary methods**. Click here for file

Additional file 2 **Statistical results for gene expression analysis**. Click here for file

Additional file 3 **Functional categories enriched among genes associated with regional emphysema severity**. Click here for file

Additional file 4 **Gene expression relevance network**. Dark blue circles are genes that have expression significantly correlated with Lm; light blue circles are all other genes. Edges are indicated by green (positive correlation) or red (negative correlation) lines. Click here for file

Additional file 5 **RT-PCR validation of 14 genes associated with regional emphysema severity**. Click here for file

Additional file 6 **Confirmation of gene expression changes associated with regional emphysema severity (Lm) within individuals with emphysema using GSEA**. Genes associated with unscaled Lm measurements identified using all eight patients in the analysis are concordantly enriched among genes associated with scaled Lm measurements (Z-scored within each patient) using only the five emphysema patients (FDR <0.001). These results demonstrate that the 127 gene signature is related to regional emphysema severity within individuals and not to differences between donors and COPD patients or to differences in levels of emphysema between COPD patients. Orange and blue color bars represent the t-statistics from correlations of gene expression with Lm. The vertical black lines represent the position of genes in the gene set among the ranked gene list. The length of the black lines corresponds to the magnitude of the running enrichment score from GSEA. Click here for file

Additional file 7 **Relation between gene expression changes associated with regional emphysema severity (Lm) and studies of TGFβ-related gene expression using GSEA**. Genes associated with Lm are enriched among the genes that are differentially expressed in response to TGFβ treatment in datasets from **(a) **Classen *et al*.[20], **(b) **Koinuma *et al*.[22], and **(c) **Malizia *et al*.[23]. **(d) **Genes most induced by TGFβ in seven studies [19-23,30,31] are enriched among the genes that are associated with Lm. Orange and blue color bars represent the t-statistics from correlations of gene expression with a continuous variable. Red and green color bars represent the fold change between samples treated with and without TGFβ. The vertical black lines represent the position of genes in the gene set among the ranked gene list. The length of the black lines corresponds to the magnitude of the running enrichment score from GSEA. Enrichments with an FDR q-value <0.05 were considered significant. Click here for file

Additional file 8 **Relation between gene expression changes associated with regional emphysema severity (Lm) and gene expression changes that occur with treatment of GHK or TGFβ in fibroblast cell lines using GSEA**. **(a) **Genes increasing in expression in response to treatment with GHK or TGFβ are enriched among genes that decrease with increasing emphysema severity. **(b) **Genes differentially expressed with TGFβ treatment or in response to GHK in the Connectivity Map are enriched among genes that change in expression with GHK (0.1 nM) in fibroblast cell lines. **(c) **Genes that are differentially expressed with TGFβ treatment or that are down-regulated with increasing emphysema severity are enriched among genes that change in expression with GHK (10 nM) in fibroblast cell lines. **(d) **Genes that are differentially expressed in response to GHK are concordantly enriched among genes that change in expression with TGFβ treatment in fibroblast cell lines. Orange and blue color bars represent the t-statistics from correlations of gene expression with a continuous variable. Red and green color bars represent the t-statistic between treated and untreated samples. The vertical black lines represent the position of genes among the ranked gene list. The length of the black lines corresponds to the magnitude of the running enrichment score from GSEA. Enrichments with an FDR q-value <0.05 were considered significant. Click here for file

Additional file 9 **Localization of members of the TGFβ superfamily using immunohistochemistry**. Representative images of positive SMAD2 staining (arrows) in the **(a) **alveolar and **(b) **small airway wall tissue. **(c) **Representative image of positive SMAD6 staining in vascular endothelial cells (arrows) and macrophages (arrowheads). **(d) **Representative image of weak SMAD1 staining in vascular endothelial cells (arrows). Representative images are shown for control IgG staining in the **(e) **alveolar wall tissue, **(f) **airway wall tissue, and **(g) **blood vessels. Scale bar = 200 µm. Click here for file

Additional file 10 **Relation between gene expression changes associated with regional emphysema severity (Lm) and cross-sectional studies of COPD-related gene expression using GSEA**. Genes associated with Lm are enriched among the genes found to associated with the presence of COPD or degree of airflow obstruction in datasets from **(a) **Golpon *et al*.[6], **(b) **Spira *et al*.[9], **(c) **Wang *et al*.[10], and **(d) **Bhattacharya *et al*.[7]. **(e) **Genes previously found to be associated with COPD-related clinical variables [6,8-10] are enriched among the genes associated with Lm. Orange and blue color bars represent the t-statistics from correlations of gene expression with a continuous variable. Red and green color bars represent the t-statistic from a *t*-test between cases and controls. The vertical black lines represent the position of genes in the gene set among the ranked gene list. The length of the black lines corresponds to the magnitude of the running enrichment score from GSEA. Enrichments with an FDR q-value <0.05 were considered significant. Click here for file

Additional file 11 **Examples of genes associated with Lm and validated by quantitative RT-PCR that were also differentially expressed in other COPD-related gene-expression datasets**. Genes such as *ACVRL1, SMAD6, CCR7, *and *CXCL13 *were associated with increasing regional emphysema severity and concordantly differentially expressed in other datasets such as Golpon *et al*.[6] and/or Wang *et al*.[10]. Click here for file

Additional file 12 **Subject demographics for lung fibroblast cultures**. Click here for file

## References

[B1] Miniño AM Xu J Kochanek KD Statistics V Deaths: Preliminary Data for 2008. National Vital Statistics Reports Volume 59 2010 NVSS http://www.cdc.gov/nchs/data/nvsr/nvsr59/nvsr59_02.pdf 25073655

[B2] Lange P Scharling H Fabricius P Vestbo J Developing COPD: a 25 year follow up study of the general population. Thorax 2006 4 935 939 10.1136/thx.2006.062802 17071833 PMC2121175

[B3] Park JW Ryter SW Choi AMK Functional significance of apoptosis in chronic obstructive pulmonary disease. COPD 2007 4 347 353 10.1080/15412550701603775 18027162

[B4] Postma DS Timens W Remodeling in asthma and chronic obstructive pulmonary disease. Proc Am Thorac Soc 2006 4 434 439 10.1513/pats.200601-006AW 16799088

[B5] Rennard SI Bailey KL Chronic obstructive pulmonary disease exacerbations: accurate and easy measurement promises much. Am J Respir Crit Care Med 2012 4 1139 1141 10.1164/rccm.201202-0227ED 22661519 PMC5448605

[B6] Golpon HA Coldren CD Zamora MR Cosgrove GP Moore MD Tuder RM Geraci MW Voelkel NF Emphysema lung tissue gene expression profiling. Am J Respir Cell Mol Biol 2004 4 595 600 10.1165/rcmb.2004-0008OC 15284076

[B7] Bhattacharya S Srisuma S Demeo DL Shapiro SD Bueno R Silverman EK Reilly JJ Mariani TJ Molecular biomarkers for quantitative and discrete COPD phenotypes. Am J Respir Cell Mol Biol 2009 4 359 367 10.1165/rcmb.2008-0114OC 18849563 PMC2645534

[B8] Ning W Li C-J Kaminski N Feghali-Bostwick C Alber SM Di YP Otterbein SL Song R Hayashi S Zhou Z Pinsky DJ Watkins SC Pilewski JM Sciurba FC Peters DG Hogg JC Choi AMK Comprehensive gene expression profiles reveal pathways related to the pathogenesis of chronic obstructive pulmonary disease. Proc Natl Acad Sci USA 2004 4 14895 14900 10.1073/pnas.0401168101 15469929 PMC522001

[B9] Spira A Beane J Pinto-Plata V Kadar A Liu G Shah V Celli B Brody JS Gene expression profiling of human lung tissue from smokers with severe emphysema. Am J Respir Cell Mol Biol 2004 4 601 610 10.1165/rcmb.2004-0273OC 15374838

[B10] Wang I-M Stepaniants S Boie Y Mortimer JR Kennedy B Elliott M Hayashi S Loy L Coulter S Cervino S Harris J Thornton M Raubertas R Roberts C Hogg JC Crackower M O'Neill G Paré PD Gene expression profiling in patients with chronic obstructive pulmonary disease and lung cancer. Am J Respir Crit Care Med 2008 4 402 411 10.1164/rccm.200703-390OC 17975202

[B11] Francis SMS Larsen JE Pavey SJ Bowman RV Hayward NK Fong KM Yang I Expression profiling identifies genes involved in emphysema severity. Respir Res 2009 4 81 10.1186/1465-9921-10-81 19723343 PMC2746189

[B12] McDonough JE Yuan R Suzuki M Seyednejad N Elliott WM Sanchez PG Wright AC Gefter WB Litzky L Coxson HO Paré PD Sin DD Pierce RA Woods JC McWilliams AM Mayo JR Lam SC Cooper JD Hogg JC Small-airway obstruction and emphysema in chronic obstructive pulmonary disease. N Engl J Med 2011 4 1567 1575 10.1056/NEJMoa1106955 22029978 PMC3238466

[B13] Lamb J Crawford ED Peck D Modell JW Blat IC Wrobel MJ Lerner J Brunet J-P Subramanian A Ross KN Reich M Hieronymus H Wei G Armstrong S Haggarty SJ Clemons P Wei R Carr S Lander ES Golub TR The Connectivity Map: using gene-expression signatures to connect small molecules, genes, and disease. Science 2006 4 1929 1935 10.1126/science.1132939 17008526

[B14] Choong CK Haddad FJ Martinez C Hu DZ Pierce J Meyers BF Patterson GA Cooper JD A simple, reproducible, and inexpensive technique in the preparation of explanted emphysematous lungs for ex vivo studies. J Thorac Cardiovasc Surg 2005 4 922 923 16153966 10.1016/j.jtcvs.2005.03.046

[B15] Zhang X Liu G Lenburg ME Spira A Comparison of smoking-induced gene expression on Affymetrix Exon and 3'-based expression arrays. Genome Inform 2007 4 247 257 18546492

[B16] Dennis G Sherman BT Hosack D Yang J Gao W Lane HC Lempicki R DAVID: Database for Annotation, Visualization, and Integrated Discovery. Genome Biol 2003 4 P3 10.1186/gb-2003-4-5-p3 12734009 PMC193660

[B17] Gentleman RC Carey VJ Bates DM Bolstad B Dettling M Dudoit S Ellis B Gautier L Ge Y Gentry J Hornik K Hothorn T Huber W Iacus S Irizarry R Leisch F Li C Maechler M Rossini AJ Sawitzki G Smith C Smyth G Tierney L Yang JYH Zhang J Bioconductor: open software development for computational biology and bioinformatics. Genome Biol 2004 4 R80 10.1186/gb-2004-5-10-r80 15461798 PMC545600

[B18] Subramanian A Tamayo P Mootha VK Mukherjee S Ebert BL Gillette M Paulovich A Pomeroy SL Golub TR Lander ES Mesirov JP Gene set enrichment analysis: a knowledge-based approach for interpreting genome-wide expression profiles. Proc Natl Acad Sci USA 2005 4 15545 15550 10.1073/pnas.0506580102 16199517 PMC1239896

[B19] Qin H Chan MWY Liyanarachchi S Balch C Potter D Souriraj IJ Cheng ASL Agosto-Perez FJ Nikonova EV Yan PS Lin H-J Nephew KP Saltz JH Showe LC Huang THM Davuluri RV An integrative ChIP-chip and gene expression profiling to model SMAD regulatory modules. BMC Syst Biol 2009 4 73 10.1186/1752-0509-3-73 19615063 PMC2724489

[B20] Classen S Zander T Eggle D Chemnitz JM Brors B Büchmann I Popov A Beyer M Eils R Debey S Schultze JL Human resting CD4+ T cells are constitutively inhibited by TGF beta under steady-state conditions. J Immunol 2007 4 6931 6940 17513742 10.4049/jimmunol.178.11.6931

[B21] Renzoni E Abraham DJ Howat S Shi-Wen X Sestini P Bou-Gharios G Wells AU Veeraraghavan S Nicholson AG Denton CP Leask A Pearson JD Black CM Welsh KI du Bois RM Gene expression profiling reveals novel TGFbeta targets in adult lung fibroblasts. Respir Res 2004 4 24 10.1186/1465-9921-5-24 15571627 PMC538264

[B22] Koinuma D Tsutsumi S Kamimura N Taniguchi H Miyazawa K Sunamura M Imamura T Miyazono K Aburatani H Chromatin immunoprecipitation on microarray analysis of Smad2/3 binding sites reveals roles of ETS1 and TFAP2A in transforming growth factor beta signaling. Mol Cell Biol 2009 4 172 186 10.1128/MCB.01038-08 18955504 PMC2612478

[B23] Malizia AP Keating DT Smith SM Walls D Doran PP Egan JJ Alveolar epithelial cell injury with Epstein-Barr virus upregulates TGFbeta1 expression. Am J Physiol Lung Cell Mol Physiol 2008 4 L451 460 10.1152/ajplung.00376.2007 18621908

[B24] Noordhoek JA Postma DS Chong LL Menkema L Kauffman HF Timens W van Straaten JFM van der Geld YM Different modulation of decorin production by lung fibroblasts from patients with mild and severe emphysema. COPD 2005 4 17 25 10.1081/COPD-200050678 17136957

[B25] Pechkovsky DV Hackett TL An SS Shaheen F Murray L Knight D Human lung parenchyma but not proximal bronchi produces fibroblasts with enhanced TGF-beta signaling and alpha-SMA expression. Am J Respir Cell Mol Biol 2010 4 641 651 10.1165/rcmb.2009-0318OC 20061511

[B26] Hackett T-L Warner SM Stefanowicz D Shaheen F Pechkovsky DV Murray L Argentieri R Kicic A Stick SM Bai TR Knight D Induction of epithelial-mesenchymal transition in primary airway epithelial cells from patients with asthma by transforming growth factor-beta1. Am J Respir Crit Care Med 2009 4 122 133 10.1164/rccm.200811-1730OC 19406982

[B27] Abraham T Carthy J McManus B Collagen matrix remodeling in 3-dimensional cellular space resolved using second harmonic generation and multiphoton excitation fluorescence. J Struct Biol 2010 4 36 44 10.1016/j.jsb.2009.07.023 19651220

[B28] Bignon J Khoury F Even P Andre J Brouet G Morphometric study in chronic obstructive bronchopulmonary disease. Pathologic, clinical, and physiologic correlations. Am Rev Respir Dis 1969 4 669 695 5772055 10.1164/arrd.1969.99.5.669

[B29] Faith JJ Hayete B Thaden JT Mogno I Wierzbowski J Cottarel G Kasif S Collins JJ Gardner TS Large-scale mapping and validation of Escherichia coli transcriptional regulation from a compendium of expression profiles. PLoS Biol 2007 4 e8 10.1371/journal.pbio.0050008 17214507 PMC1764438

[B30] Chambers RC Leoni P Kaminski N Laurent GJ Heller RA Global expression profiling of fibroblast responses to transforming growth factor-beta1 reveals the induction of inhibitor of differentiation-1 and provides evidence of smooth muscle cell phenotypic switching. Am J Pathol 2003 4 533 546 10.1016/S0002-9440(10)63847-3 12547711 PMC1851161

[B31] Verrecchia F Chu ML Mauviel A Identification of novel TGF-beta/Smad gene targets in dermal fibroblasts using a combined cDNA microarray/promoter transactivation approach. J Biol Chem 2001 4 17058 17062 10.1074/jbc.M100754200 11279127

[B32] Siméon A Wegrowski Y Bontemps Y Maquart FX Expression of glycosaminoglycans and small proteoglycans in wounds: modulation by the tripeptide-copper complex glycyl-L-histidyl-L-lysine-Cu(2+). J Invest Dermatol 2000 4 962 968 10.1046/j.1523-1747.2000.00166.x 11121126

[B33] Maquart FX Bellon G Chaqour B Wegrowski J Patt LM Trachy RE Monboisse JC Chastang F Birembaut P Gillery P In vivo stimulation of connective tissue accumulation by the tripeptide-copper complex glycyl-L-histidyl-L-lysine-Cu2+ in rat experimental wounds. J Clin Invest 1993 4 2368 2376 10.1172/JCI116842 8227353 PMC288419

[B34] Pickart L The human tri-peptide GHK and tissue remodeling. J Biomater Sci Polym Ed 2008 4 969 988 10.1163/156856208784909435 18644225

[B35] Togo S Holz O Liu X Sugiura H Kamio K Wang X Kawasaki S Ahn Y Fredriksson K Skold CM Mueller KC Branscheid D Welker L Watz H Magnussen H Rennard SI Lung fibroblast repair functions in patients with chronic obstructive pulmonary disease are altered by multiple mechanisms. Am J Respir Crit Care Med 2008 4 248 260 10.1164/rccm.200706-929OC 18467512 PMC2542423

[B36] Hogg JC Chu F Utokaparch S Woods R Elliott WM Buzatu L Cherniack RM Rogers RM Sciurba FC Coxson HO Paré PD The nature of small-airway obstruction in chronic obstructive pulmonary disease. N Engl J Med 2004 4 2645 2653 10.1056/NEJMoa032158 15215480

[B37] Brusselle GG Demoor T Bracke KR Brandsma C-a Timens W Lymphoid follicles in (very) severe COPD: beneficial or harmful?. Eur Respir J 2009 4 219 230 10.1183/09031936.00150208 19567605

[B38] van der Strate BW Postma DS Brandsma C-A Melgert BN Luinge M Geerlings M Hylkema MN van den Berg A Timens W Kerstjens HaM Cigarette smoke-induced emphysema: A role for the B cell?. Am J Respir Crit Care Med 2006 4 751 758 10.1164/rccm.200504-594OC 16399994

[B39] Blobe GC Schiemann WP Lodish HF Role of transforming growth factor beta in human disease. N Engl J Med 2000 4 1350 1358 10.1056/NEJM200005043421807 10793168

[B40] Morty RE Königshoff M Eickelberg O Transforming growth factor-beta signaling across ages: from distorted lung development to chronic obstructive pulmonary disease. Proc Am Thorac Soc 2009 4 607 613 10.1513/pats.200908-087RM 19934357

[B41] Zandvoort A Postma DS Jonker MR Noordhoek J Vos JTWM van der Geld YM Timens W Altered expression of the Smad signalling pathway: implications for COPD pathogenesis. Eur Respir J 2006 4 533 541 10.1183/09031936.06.00078405 16707515

[B42] Zandvoort A Postma DS Jonker MR Noordhoek J Vos JTWM Timens W Smad gene expression in pulmonary fibroblasts: indications for defective ECM repair in COPD. Respir Res 2008 4 83 10.1186/1465-9921-9-83 19087346 PMC2613883

[B43] Springer J Scholz FR Peiser C Groneberg D Fischer A SMAD-signaling in chronic obstructive pulmonary disease: transcriptional down-regulation of inhibitory SMAD 6 and 7 by cigarette smoke. Biol Chem 2004 4 649 653 15318814 10.1515/BC.2004.080

[B44] Leppäranta O Myllärniemi M Salmenkivi K Kinnula VL Keski-Oja J Koli K Reduced phosphorylation of the TGF-Beta signal transducer Smad2 in emphysematous human lung. COPD 2009 4 234 241 10.1080/15412550903049173 19811381

[B45] Gosselink JV Hayashi S Elliott WM Xing L Chan B Yang L Wright C Sin D Paré PD Pierce J Pierce R Patterson A Cooper J Hogg JC Differential expression of tissue repair genes in the pathogenesis of chronic obstructive pulmonary disease. Am J Respir Crit Care Med 2010 4 1329 1335 10.1164/rccm.200812-1902OC 20075389 PMC2894408

[B46] Smolonska J Wijmenga C Postma DS Boezen HM Meta-analyses on suspected chronic obstructive pulmonary disease genes: a summary of 20 years' research. Am J Respir Crit Care Med 2009 4 618 631 10.1164/rccm.200905-0722OC 19608716

[B47] van Diemen CC Postma DS Vonk JM Bruinenberg M Nolte IM Boezen HM Decorin and TGF-beta1 polymorphisms and development of COPD in a general population. Respir Res 2006 4 89 10.1186/1465-9921-7-89 16780585 PMC1539000

[B48] van Diemen CC Postma DS Aulchenko YS Snijders PJLM Oostra B van Duijn CM Boezen HM Novel strategy to identify genetic risk factors for COPD severity: a genetic isolate. Eur Respir J 2010 4 768 775 10.1183/09031936.00054408 19797132

[B49] Dudley JT Sirota M Shenoy M Pai RK Roedder S Chiang AP Morgan A Sarwal MM Pasricha PJ Butte AJ Computational repositioning of the anticonvulsant topiramate for inflammatory bowel disease. Sci Transl Med 2011 4 96ra76 10.1126/scitranslmed.3002648 21849664 PMC3479650

[B50] Sirota M Dudley JT Kim J Chiang AP Morgan A Sweet-Cordero A Sage J Butte AJ Discovery and preclinical validation of drug indications using compendia of public gene expression data. Sci Transl Med 2011 4 96ra77 10.1126/scitranslmed.3001318 21849665 PMC3502016

[B51] Kang Y-A Choi H-R Na J-I Huh C-H Kim M-J Youn S-W Kim K-H Park K-C Copper-GHK increases integrin expression and p63 positivity by keratinocytes. Arch Dermatol Res 2009 4 301 306 10.1007/s00403-009-0942-x 19319546

