# Supplementary material for: A gene expression signature of emphysema-related lung destruction and its reversal by the tripeptide GHK
Source: Genome Med. 2012 Aug 31;4(8):67. doi: 10.1186/gm367 (PMC4064320; doi:10.1186/gm367)
Supplement: Additional file 1 — Supplementary methods. [file gm368-S1.DOC]

Additional File 1: Supplementary Methods

**Reverse Engineering of Transcriptional Networks.** CLR scores between all possible pairs of genes were computed using CLR version 1.2 [29], and a significance cut-off of FDR q-value < 2.0 x 10^-5^ was selected. The CLR algorithm calculates these scores by computing mutual information between all possible pairs of genes and then applying a z-score correction. A sub-network was created consisting of edges between differentially expressed transcription factors and genes connected to these transcription factors and edges between non-differentially expressed transcription factors connected to differentially expressed genes. The direction of the correlation was determined by the sign of a Pearson correlation.

**Connecting to other gene-expression datasets.** Using GSEA, sets of genes that change with COPD-related phenotypes or with TGFβ treatment in other gene-expression studies were compared to a ranked list of genes ordered by the t-statistic of the β_Lm_ coefficient in the linear mixed-effects model. Conversely, the sets of genes positively and negatively correlated to Lm were compared to ranked genes lists generated by re-analyzing gene-expression datasets from these additional COPD- and TGFβ-related studies. The top 50 up- and down-regulated genes from the ranked gene lists for these studies were used as query signatures in the Connectivity Map. Below is a description of the data normalization procedures and statistical analyses used to generate gene sets and ranked gene lists for each gene-expression dataset:

Golpon (GSE1122)

CEL files were obtained from GEO and normalized with RMA using the custom Entrez Gene CDF v11.0.1. Two ranked gene lists were generated using the t-statistic from a t-test comparing patients with emphysema (n=5) vs. controls (n=5) or α1-antitrypsin deficient patients (n=5) vs. controls (n=5). Sets of genes up- or down-regulated with emphysema were retrieved from the original publication.

Wang (GSE8500)

Preprocessed gene-expression data, clinical characteristics for each sample, and the corresponding gene symbol for each probeset were retrieved from GEO. Three ranked gene lists were generated using the t-statistic from a Pearson correlation comparing gene expression profiles to FEV_1_ percent predicted (n=48), FEV_1_/FVC (n=48), or DLCO (n=47). In addition, two ranked gene lists were generated using the t-statistic from t-tests comparing GOLD2 patients (n=10) vs. non-smokers (n=5) or GOLD3 patients (n=3) vs. non-smokers (n=5). Sets of genes positively or negatively correlated with FEF(25-75%) were retrieved from the original publication.

Bhattacharya (GSE8581)

CEL files were obtained from GEO and normalized with RMA using the custom Entrez Gene CDF v11.0.1. One sample was an outlier in a PCA and was filtered out from further analysis. Two ranked gene lists were generated using the t-statistic from a Pearson correlation comparing gene expression profiles to FEV_1_ percent predicted (n=57) or FEV_1_/FVC (n=57). In addition, a ranked gene list was generated using the t-statistic from a t-test comparing COPD (n=15) vs. controls (n=19) as defined in the original study. Sets of genes up- or down-regulated between cases vs. controls and sets of genes positively or negatively correlated with FEV_1_ percent predicted or FEV_1_/FVC were retrieved from the original publication.

Spira (GSE1650)

CEL files were obtained from GEO and normalized with RMA using the custom Entrez Gene CDF v11.0.1. Three ranked gene lists were generated using the t-statistic from a Pearson correlation comparing gene expression profiles to FEV_1_ percent predicted (n=30), FEV_1_/FVC (n=22), or DLCO (n=28). In addition, a ranked gene list was generated using the t-statistic from a t-test comparing COPD (n=18) vs. controls (n=12) as defined in the original study. Sets of genes up- or down-regulated between cases vs. controls and sets of genes positively or negatively correlated with FEV_1_ percent predicted, DLCO, or BMI were retrieved from the original publication.

Francis (GSE17770)

Preprocessed gene-expression data, clinical characteristics for each sample, and the corresponding gene symbol for each probeset were retrieved from GEO. Two ranked gene lists were generated using the t-statistic from a Pearson correlation comparing gene expression profiles to FEV_1_ percent predicted (n=30) or KCO percent predicted (n=30). Sets of genes positively or negatively correlated with KCO percent predicted were retrieved from the original publication.

Ning

The set of genes up- or down-regulated between GOLD2 vs.GOLD0 patients were retrieved from the original publication. No ranked gene lists were generated as the microarray data was not publicly available.

Chambers

The set of genes up-regulated by TGFβ treatment in human fetal lung fibroblasts at any time point were retrieved from the original publication. No ranked gene lists were generated as the microarray data was not publicly available.

Verrecchia

The set of genes up-regulated by TGFβ treatment in human dermal fibroblasts were reported in the original publication and retrieved from MSigDB 2.5. No ranked gene lists were generated as the microarray data was not publicly available.

Classen (GSE7497)

Preprocessed gene-expression data and the corresponding gene symbol for each probeset were retrieved from GEO. Four ranked gene lists were generated using fold changes between serum deprived CD4+ T cells treated with (n=3) and without (n=3) TGFβ1 for four different time points. Gene sets were generated by taking the top 200 genes up-regulated and the top 200 genes down-regulated with TGFβ treatment.

Malizia (GSE5450)

CEL files were obtained from GEO and normalized with RMA using the custom Entrez Gene CDF v11.0.1. A ranked gene list was generated using fold changes between A549 cells treated with (n=2) and without (n=2) TGFβ. Gene sets were generated by taking the top 200 genes up-regulated and the top 200 genes down-regulated with TGFβ treatment.

Qin (GSE6653)

CEL files were obtained from GEO and normalized with RMA using the custom Entrez Gene CDF v11.0.1. Three ranked gene lists were generated using fold changes between immortalized ovarian surface epithelial cells treated with (n=2) and without (n=2) TGFβ for three different time points. Sets of genes up- or down-regulated with TGFβ treatment were retrieved from the original publication.

Koinuma (GSE11710)

CEL files were obtained from GEO and normalized with RMA using the custom Entrez Gene CDF v11.0.1. Three ranked gene lists were generated using fold changes between HaCaT keratinocytes treated with (n=1) and without (n=1) TGFβ for three different time points. Gene sets were generated by taking the top 200 genes up-regulated and the top 200 genes down-regulated with TGFβ treatment.

Renzoni (GSE1724)

Preprocessed gene-expression data was retrieved from GEO. A ranked gene list was generated using fold changes between control lung fibroblasts treated with (n=3) and without (n=3) TGFβ. Gene sets were generated by taking the top 200 genes up-regulated and the top 200 genes down-regulated with TGFβ treatment.

**Real time PCR validation.** Quantitative RT-PCR analysis was used to confirm the expression levels of select genes. ACVRL1, BCL11A, CCR7, CD79A, CXCL13, EPAS1, FOXF1, KLF13, S100A8, SMAD6, WFDC1, and TAL1 were associated with Lm (FDR < 0.10) while GATA2 and TBX3 were among the most highly connected genes in the relevance network. Three of the subjects with severe emphysema (6965, 6969, and 6970) were used with four tissue cores per patient. Primer sequences for the fourteen genes chosen for validation were designed with PRIMER EXPRESS software (Applied Biosystems, Foster City, CA). Primer sequences to measure the expression of housekeeping genes (GAPDH, TBP, YWHAZ) were adopted from Vandesompele *et al* [52]. RNA samples (2 μg of RNA from the samples used in the microarray analysis) were treated with TURBO DNA-free (Ambion), according to the manufacturer's protocol, to remove contaminating genomic DNA. Total RNA was reverse-transcribed using random hexamers (Applied Biosystems) and SuperScript II reverse transcriptase (Invitrogen). The resulting first-strand cDNA was diluted with nuclease-free water (Ambion) to 4 ng/μl. PCR amplification mixtures (25 μl) contained 20 ng template cDNA, 12.5 μl of 2× SYBR Green PCR master mix (Applied Biosystems) and 300 nM forward and reverse primers. Forty cycles of amplification and data acquisition were carried out in StepOnePlus Real-Time PCR systems (Applied Biosystems). Threshold determinations were automatically performed by StepOne Software (version 2.0.2; Applied Biosystems) for each reaction. All real-time PCR experiments were carried out in triplicate on each sample. Data analysis was performed using geNorm [52]. Three genes (GAPDH, TBP, YWHAZ) were used for normalization.

**Immunohistochemistry.** Portions of a frozen tissue core close to the source of RNA were vacuum embedded in diluted Tissue-Tek O.C.T. compound (Sakura Finetek USA Inc) (50% vol/vol) in PBS containing 10% sucrose kept just above the freezing point and immediately refrozen on dry ice. Histology sections cut from these frozen blocks were air-dried at room temperature overnight and stained with appropriate antibodies (See the table below for antibody information). Each antibody was optimized prior to performing a staining run on an automatic immunostainer (Dako) using MACH 4 Universal AP Polymer Detection Kit (BioCare Medical), and each section was counterstained with hematoxylin. Non-specific IgG substitution for specific antibodies provided negative controls. Digital images were captured using a SPOT camera and Nikon E-800 microscope and the volume fraction of the tissue containing positively stained cells was determined using Image-Pro Plus software (Media Cybernetics) as performed previously [53].

| **Antibody** | **Company** | **Catalog No.** | **Host** | **Dilution** | **Fixation** |
| --- | --- | --- | --- | --- | --- |
| CD79a | Dako | M7050 | Mouse | 1/50 | acetone, RT,  10 min |
| SMAD1 | Abnova | DP0239 | Rabbit | 1/400 | cold acetone,  5 min |
| SMAD2 | LifeSpan Biosciences | LS-C39128 | Rabbit | 1/100 | acetone, RT,  10 min |
| SMAD6 | LifeSpan Biosciences | LS-B2065 | Rabitt | 1/25 | 10% formalin, RT,  10 min |

**Fibroblast cultures.** Normal human diploid lung fibroblasts (HFL-1) were obtained from the American Type Culture Collection (Manassas, VA) and used between 3-5 subsequent passages after thawing of frozen cells. HFL-1 cells were placed in 6-well culture plates (BD Biosciences) at a density of 1x10^5^ cells per well in complete cell culture medium (CCM), consisting of DMEM supplemented with 10% FBS, 2 mM L-glutamine and 1% of antibiotic solution (Invitrogen). Cells were cultured at 37°C and 5% CO_2_ in CCM until they reached 80% confluence. Cells were quiesced in CCM with 1% FBS overnight. HFL-1 cultures were treated with Gly-His-Lys acetate (GHK, Sigma-Aldrich) at concentrations of 100 pM and 10 nM, recombinant human TGFβ1 (10 ng/ml, PeproTech) alone for 48 hrs. Cells exposed to the CCM with added DMSO served as a vehicle control. Replicates were derived from subsequent passages of HFL-1 cell cultures (n=3). Total RNA was isolated from HFL-1 monolayers using RNeasy Plus Mini-Kit (Qiagen) as per manufacturer’s instructions. Quantity and quality of the isolated RNA was determined using Agilent 2100 Bioanalyzer (Agilent Technologies). 200 ng of total RNA was processed and hybridized onto the Human Gene 1.0 ST array (Affymetrix Inc.) as previously described [54]. Transcript-level gene expression estimates were generated using RMA with the Entrez gene CDF version v11.0.1 [55]. Differentially expressed gene expression profiles were identified using a one-way ANOVA for GHK treatment and a t-test for TGFβ1 treatment. Gene expression profiles were ranked for each treatment by t-statistic. The 200 most up-regulated and the 200 most down-regulated gene profiles were used as gene sets for each treatment in GSEA.
