# Supplementary figures and images for: A gene expression signature of emphysema-related lung destruction and its reversal by the tripeptide GHK
Source: Genome Med. 2012 Aug 31;4(8):67. doi: 10.1186/gm367 (PMC4064320; doi:10.1186/gm367)

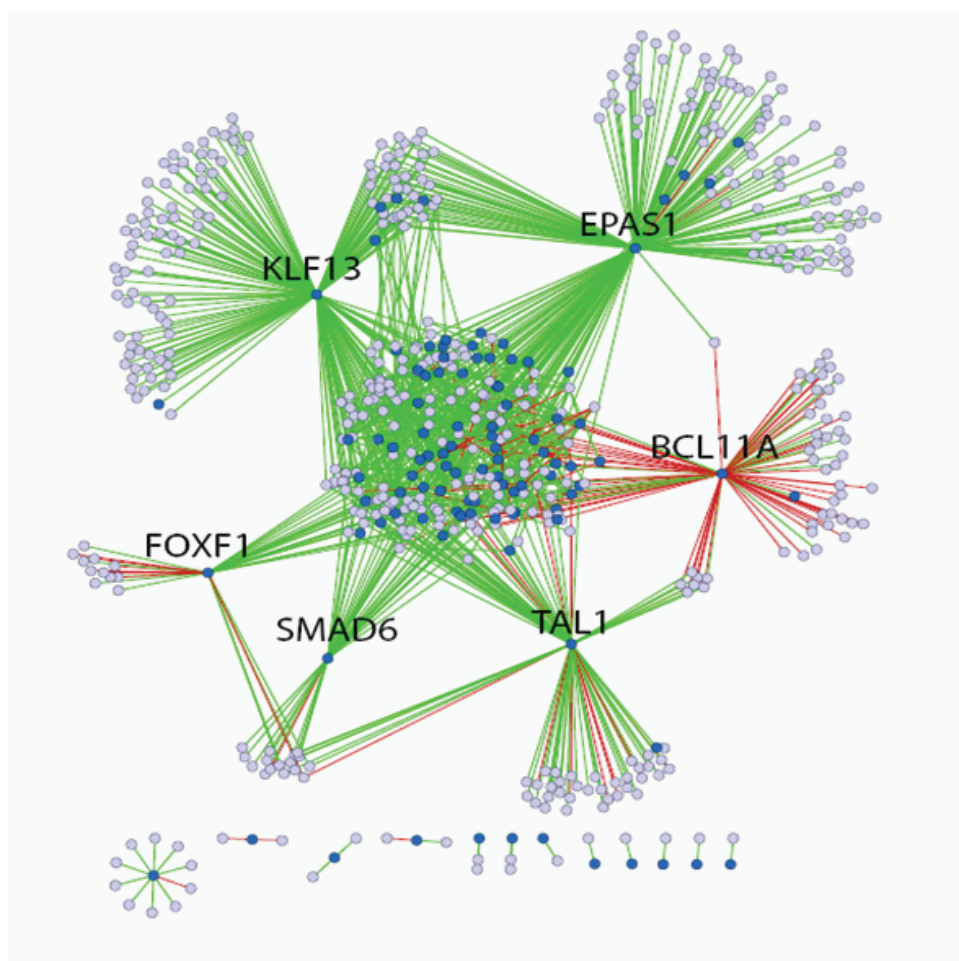

**Additional File 4. Gene expression relevance network.**

Supplement: Additional file 4 — Gene expression relevance network. Dark blue circles are genes that have expression significantly correlated with Lm; light blue circles are all other genes. Edges are indicated by green (positive correlation) or red (negative correlation) lines. [file gm368-S4.PDF]

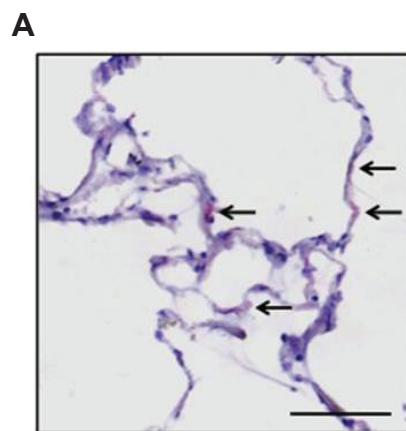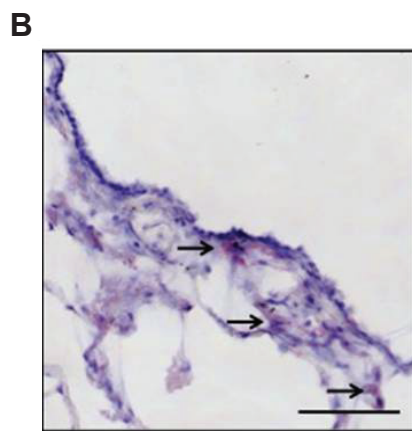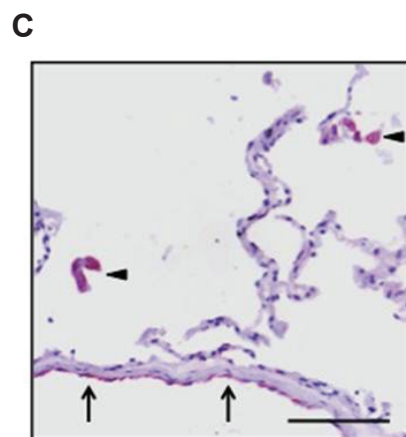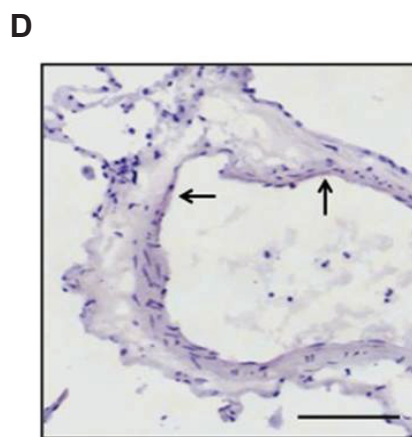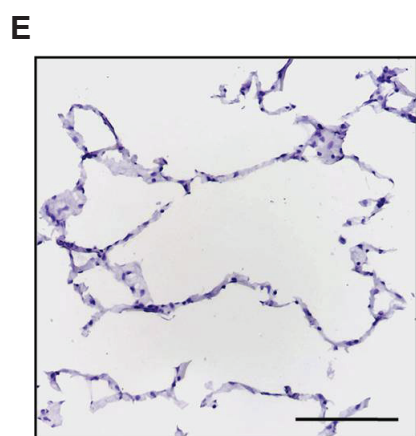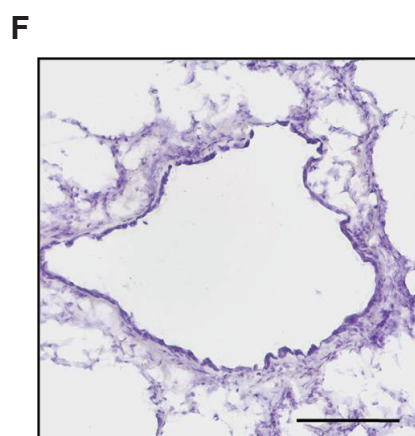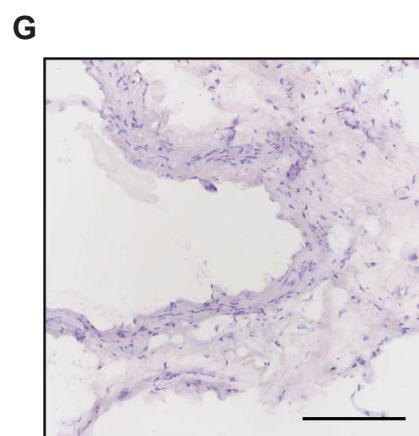

**Additional File 9. Localization of members of the TGF $\beta$  superfamily using IHC.**

Supplement: Additional file 9 — Localization of members of the TGFβ superfamily using immunohistochemistry. Representative images of positive SMAD2 staining (arrows) in the (a) alveolar and (b) small airway wall tissue. (c) Representative image of positive SMAD6 staining in vascular endothelial cells (arrows) and macrophages (arrowheads). (d) Representative image of weak SMAD1 staining in vascular endothelial cells (arrows). Representative images are shown for control IgG staining in the (e) alveolar wall tissue, (f) airway wall tissue, and (g) blood vessels. Scale bar = 200 µm. [file gm368-S9.PDF]
