# Supplementary material for: A gene expression signature of emphysema-related lung destruction and its reversal by the tripeptide GHK
Source: Genome Med. 2012 Aug 31;4(8):67. doi: 10.1186/gm367 (PMC4064320; doi:10.1186/gm367)
Supplement: Additional file 6 — Confirmation of gene expression changes associated with regional emphysema severity (Lm) within individuals with emphysema using GSEA. Genes associated with unscaled Lm measurements identified using all eight patients in the analysis are concordantly enriched among genes associated with scaled Lm measurements (Z-scored within each patient) using only the five emphysema patients (FDR <0.001). These results demonstrate that the 127 gene signature is related to regional emphysema severity within individuals and not to differences between donors and COPD patients or to differences in levels of emphysema between COPD patients. Orange and blue color bars represent the t-statistics from correlations of gene expression with Lm. The vertical black lines represent the position of genes in the gene set among the ranked gene list. The length of the black lines corresponds to the magnitude of the running enrichment score from GSEA. [file gm368-S6.PDF]

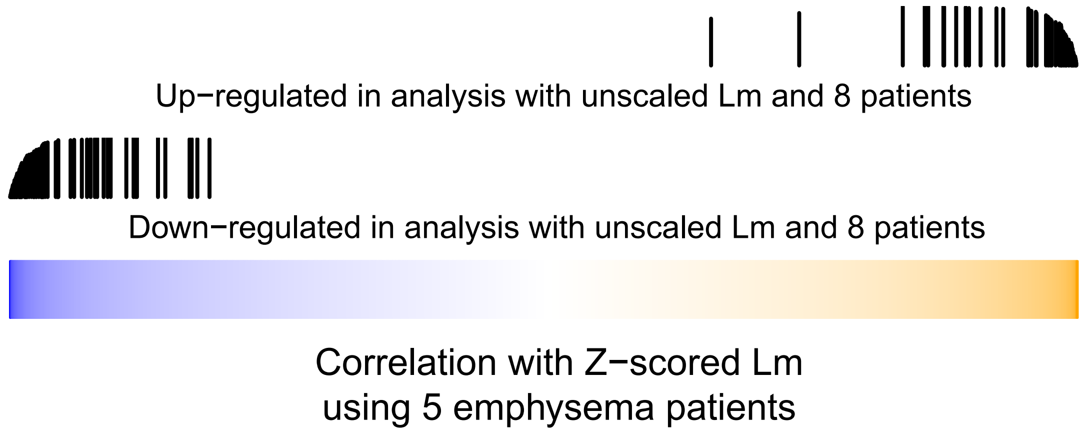

Additional File 6. Confirmation of gene expression changes associated with regional emphysema severity (Lm) within individuals with emphysema using GSEA.
