# Supplementary material for: A gene expression signature of emphysema-related lung destruction and its reversal by the tripeptide GHK
Source: Genome Med. 2012 Aug 31;4(8):67. doi: 10.1186/gm367 (PMC4064320; doi:10.1186/gm367)
Supplement: Additional file 7 — Relation between gene expression changes associated with regional emphysema severity (Lm) and studies of TGFβ-related gene expression using GSEA. Genes associated with Lm are enriched among the genes that are differentially expressed in response to TGFβ treatment in datasets from (a) Classen et al.[20], (b) Koinuma et al.[22], and (c) Malizia et al.[23]. (d) Genes most induced by TGFβ in seven studies [19-23,30,31] are enriched among the genes that are associated with Lm. Orange and blue color bars represent the t-statistics from correlations of gene expression with a continuous variable. Red and green color bars represent the fold change between samples treated with and without TGFβ. The vertical black lines represent the position of genes in the gene set among the ranked gene list. The length of the black lines corresponds to the magnitude of the running enrichment score from GSEA. Enrichments with an FDR q-value <0.05 were considered significant. [file gm368-S7.PDF]

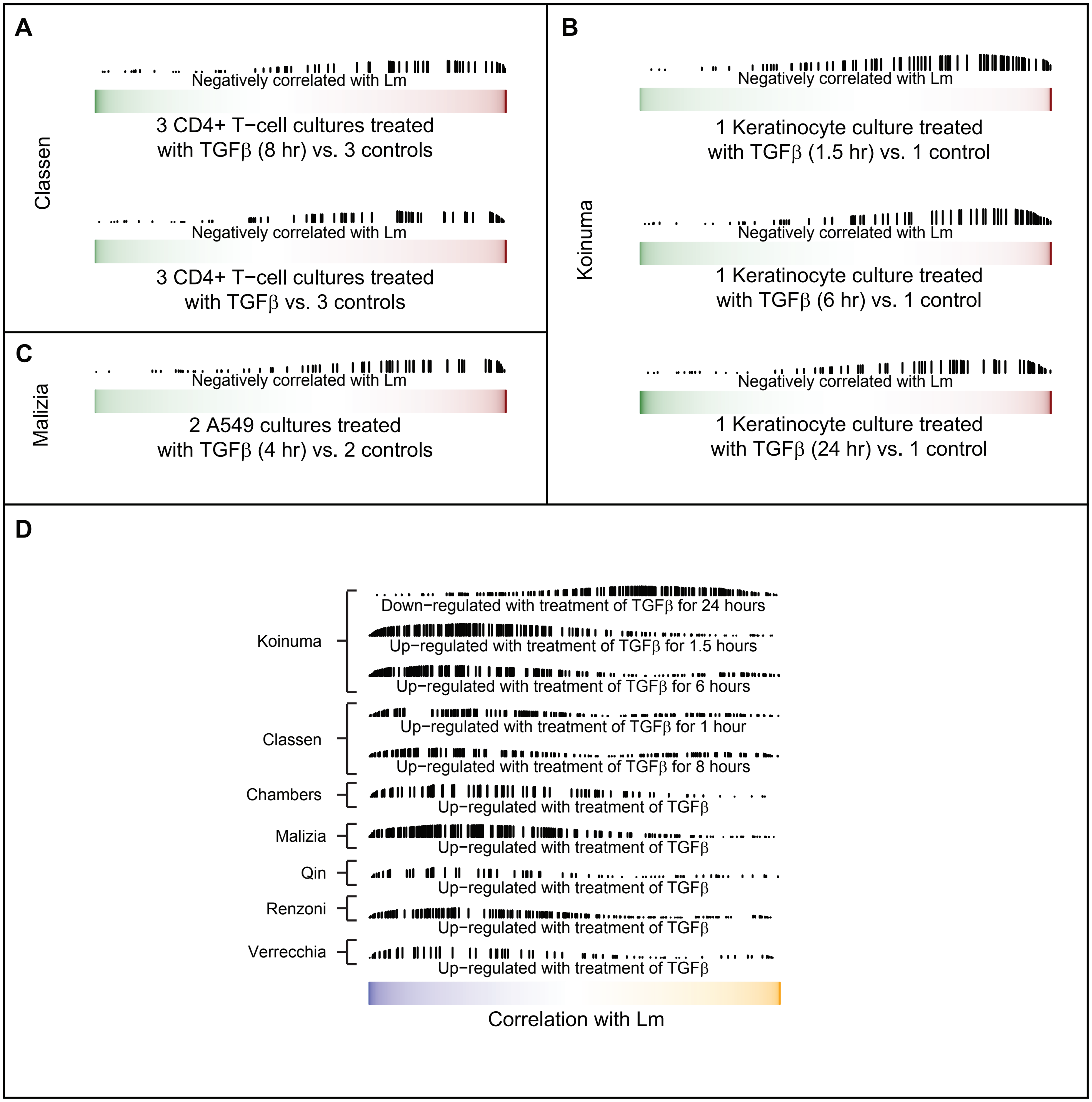

Additional File 7. Relation between gene expression changes associated with regional emphysema severity (Lm) and studies of TGFβ–related gene expression using GSEA.
