# Supplementary material for: A gene expression signature of emphysema-related lung destruction and its reversal by the tripeptide GHK
Source: Genome Med. 2012 Aug 31;4(8):67. doi: 10.1186/gm367 (PMC4064320; doi:10.1186/gm367)
Supplement: Additional file 8 — Relation between gene expression changes associated with regional emphysema severity (Lm) and gene expression changes that occur with treatment of GHK or TGFβ in fibroblast cell lines using GSEA. (a) Genes increasing in expression in response to treatment with GHK or TGFβ are enriched among genes that decrease with increasing emphysema severity. (b) Genes differentially expressed with TGFβ treatment or in response to GHK in the Connectivity Map are enriched among genes that change in expression with GHK (0.1 nM) in fibroblast cell lines. (c) Genes that are differentially expressed with TGFβ treatment or that are down-regulated with increasing emphysema severity are enriched among genes that change in expression with GHK (10 nM) in fibroblast cell lines. (d) Genes that are differentially expressed in response to GHK are concordantly enriched among genes that change in expression with TGFβ treatment in fibroblast cell lines. Orange and blue color bars represent the t-statistics from correlations of gene expression with a continuous variable. Red and green color bars represent the t-statistic between treated and untreated samples. The vertical black lines represent the position of genes among the ranked gene list. The length of the black lines corresponds to the magnitude of the running enrichment score from GSEA. Enrichments with an FDR q-value <0.05 were considered significant. [file gm368-S8.PDF]

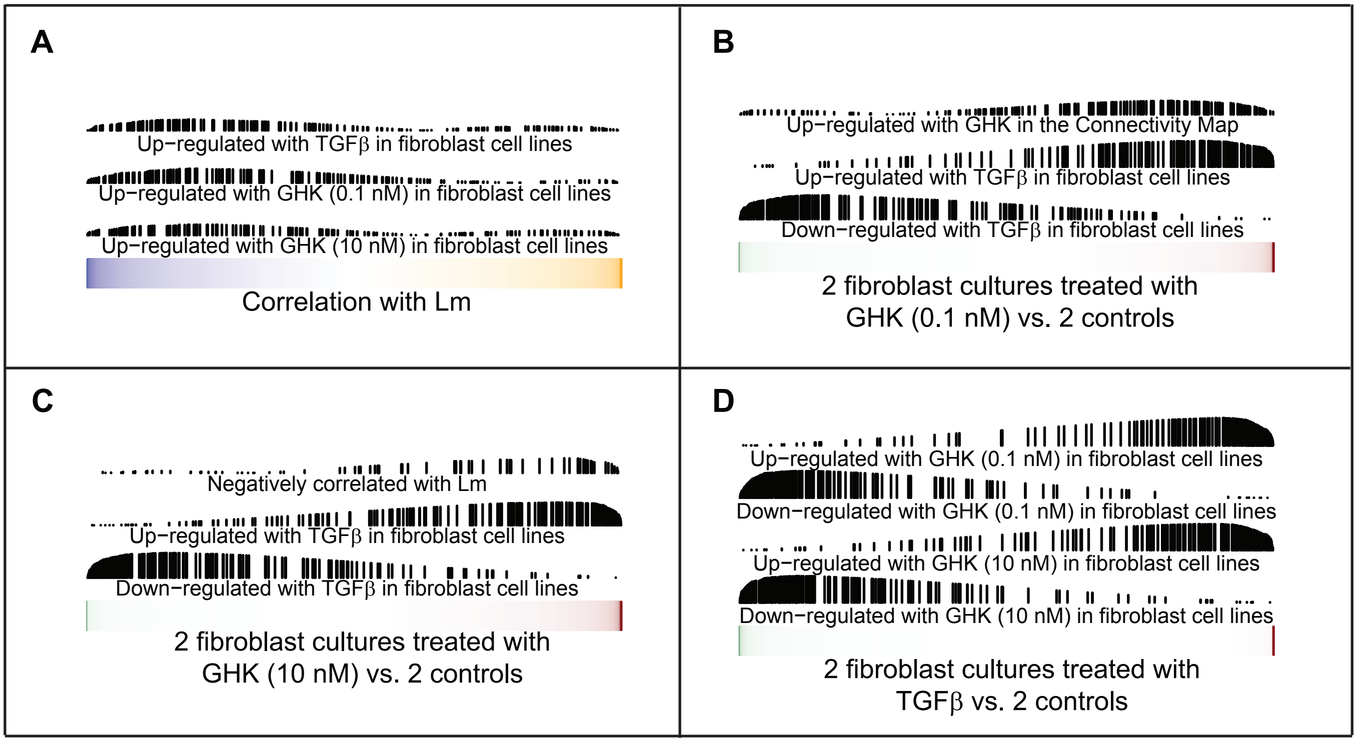

Additional File 8. Relation between gene expression changes associated with regional emphysema severity (Lm) and gene expression changes that occur with treatment of GHK or TGFβ in fibroblast cell lines using GSEA.
