# Supplementary material for: A gene expression signature of emphysema-related lung destruction and its reversal by the tripeptide GHK
Source: Genome Med. 2012 Aug 31;4(8):67. doi: 10.1186/gm367 (PMC4064320; doi:10.1186/gm367)
Supplement: Additional file 10 — Relation between gene expression changes associated with regional emphysema severity (Lm) and cross-sectional studies of COPD-related gene expression using GSEA. Genes associated with Lm are enriched among the genes found to associated with the presence of COPD or degree of airflow obstruction in datasets from (a) Golpon et al.[6], (b) Spira et al.[9], (c) Wang et al.[10], and (d) Bhattacharya et al.[7]. (e) Genes previously found to be associated with COPD-related clinical variables [6,8-10] are enriched among the genes associated with Lm. Orange and blue color bars represent the t-statistics from correlations of gene expression with a continuous variable. Red and green color bars represent the t-statistic from a t-test between cases and controls. The vertical black lines represent the position of genes in the gene set among the ranked gene list. The length of the black lines corresponds to the magnitude of the running enrichment score from GSEA. Enrichments with an FDR q-value <0.05 were considered significant. [file gm368-S10.PDF]

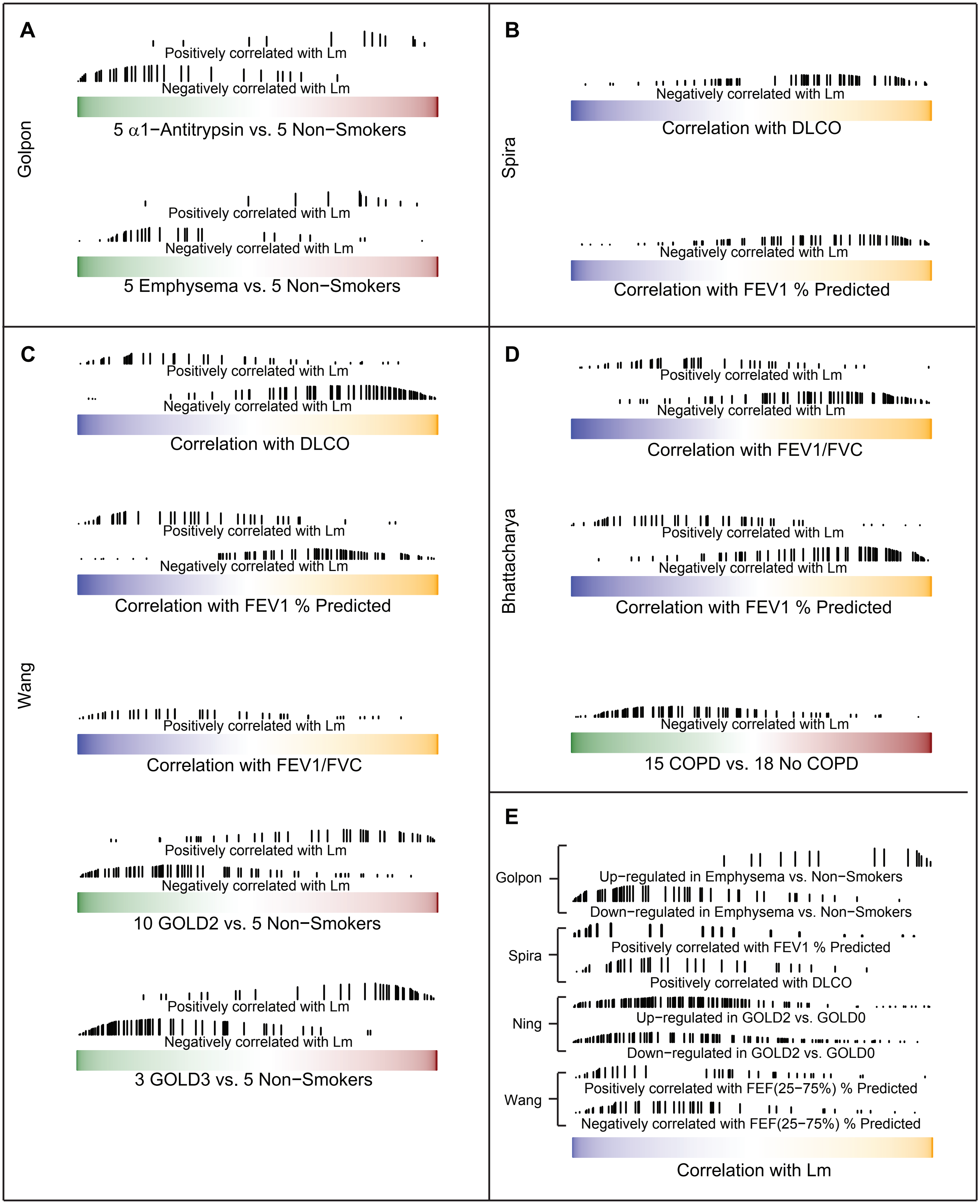

Additional File 10. Relation between gene expression changes associated with regional emphysema severity (Lm) and cross-sectional studies of COPD–related gene expression using GSEA.
