# Supplementary material for: A gene expression signature of emphysema-related lung destruction and its reversal by the tripeptide GHK
Source: Genome Med. 2012 Aug 31;4(8):67. doi: 10.1186/gm367 (PMC4064320; doi:10.1186/gm367)
Supplement: Additional file 11 — Examples of genes associated with Lm and validated by quantitative RT-PCR that were also differentially expressed in other COPD-related gene-expression datasets. Genes such as ACVRL1, SMAD6, CCR7, and CXCL13 were associated with increasing regional emphysema severity and concordantly differentially expressed in other datasets such as Golpon et al.[6] and/or Wang et al.[10]. [file gm368-S11.PDF]

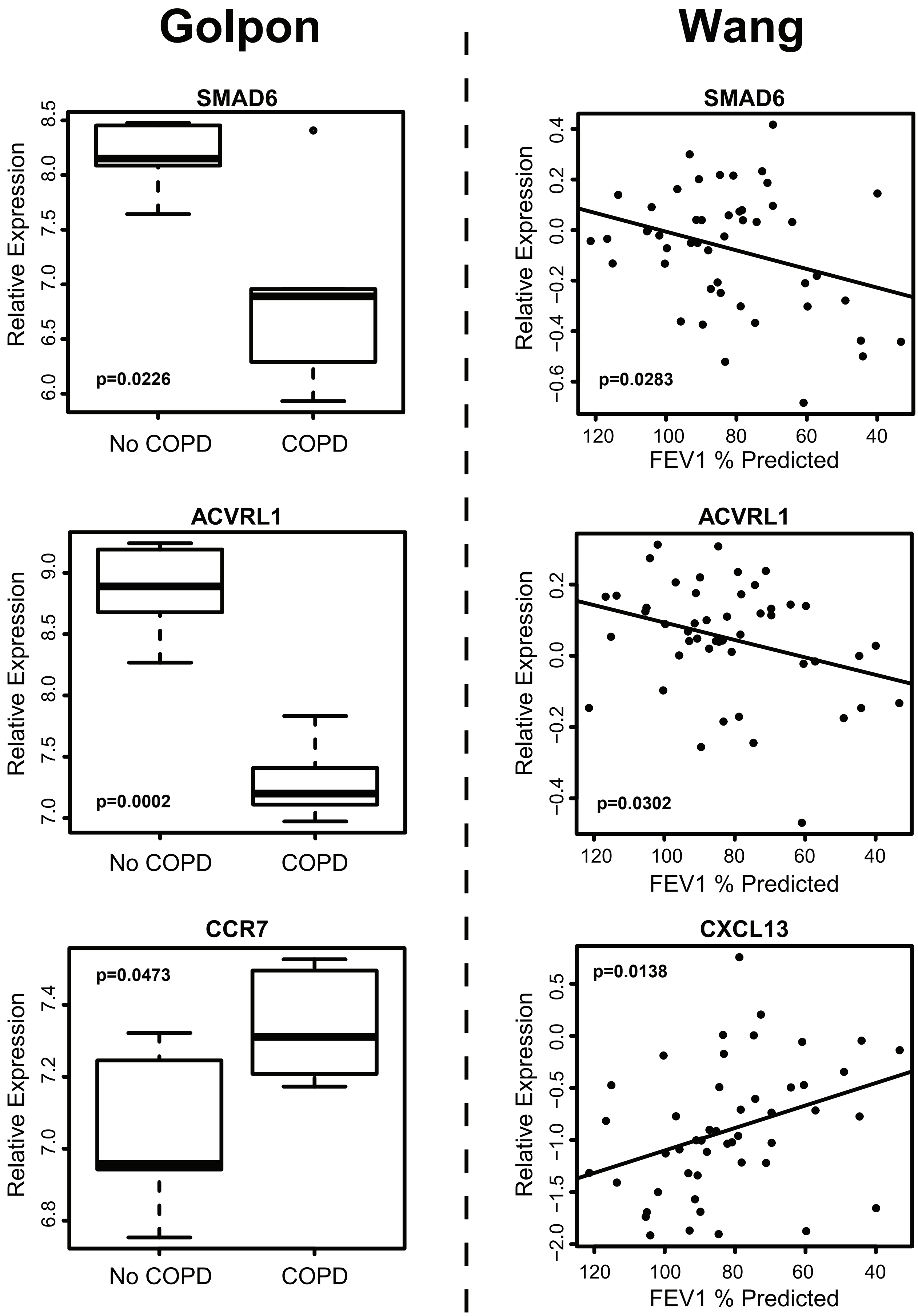

Additional File 11. Examples of genes associated with Lm and validated by qRT-PCR that were also differentially expressed in other COPD-related gene-expression datasets.
